# Degradable Alternating Copolymers by Radical Copolymerization of 2-Methylen-1,3-dioxepane and Crotonate Esters

**DOI:** 10.1021/acsmacrolett.4c00101

**Published:** 2024-03-08

**Authors:** Aitor Barquero, Arianna Zanoni, Elena Gabirondo, Estibaliz González de San Román, Shaghayegh Hamzehlou, Marta Ximenis, Davide Moscatelli, Haritz Sardon, Jose Ramon Leiza

**Affiliations:** † POLYMAT and Department of Applied Chemistry, University of the Basque Country UPV/EHU, Joxe Mari Korta Center, Tolosa hiribidea, 72, 20018 Donostia, Spain; ‡ Department of Chemistry, Materials and Chemical Engineering “Giulio Natta”, Politecnico di Milano, via Mancinelli 7, 2013 Milano, Italy; § POLYMAT and Department of Polymers and Advanced Materials/ Physics, Chemistry and Technology, Faculty of Chemistry, University of the Basque Country UPV/EHU, Paseo Manuel de Lardizabal 3, 20018 Donostia-San Sebastián, Spain; ∥ POLYMAT, University of the Basque Country UPV/EHU, Joxe Mari Korta Center, Tolosa hiribidea 72, 20018 Donostia, Spain

## Abstract

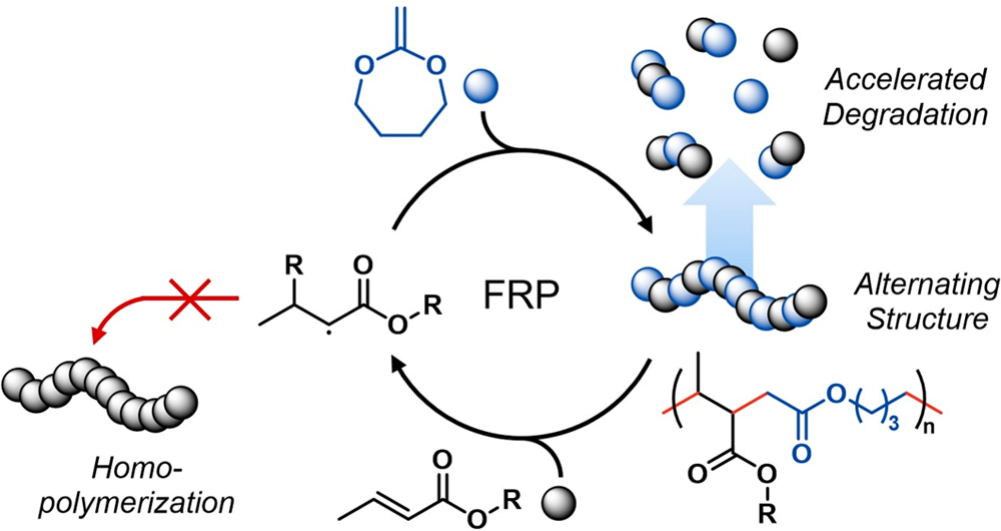

Producing backbone degradable copolymers via free-radical copolymerization is a promising, yet challenging method to develop more sustainable materials for many applications. In this work, we present the copolymerization of 2-methylen-1,3-dioxepane (MDO) with crotonic acid derivative esters. MDO can copolymerize by radical ring-opening polymerization incorporating degradable ester moieties in the polymer backbone, although this can often be difficult due to the very unfavorable reactivity ratios. Crotonic acid derivatives, on the other hand, can be easily produced completely from biomass but are typically very difficult to (co)polymerize due to low propagation rates and very unfavorable reactivity ratios. Herein, we present the surprisingly easy copolymerization between MDO and butyl crotonate (BCr), which shows the ability to form alternating copolymers. The alternating nature of the copolymer was characterized by MALDI-TOF and supported by the reactivity ratios calculated experimentally (*r*_MDO_ = 0.105 and *r*_BCr_ = 0.017). The alternating nature of the copolymers favored the degradability that could be achieved under basic conditions (in 2 h, all chains have molar masses smaller than 2 kg/mol). Last, the work was expanded to other crotonate monomers to expand the portfolio and show the potential of this copolymer family.

In the 21st century, the effects of environmental pollution and the release of greenhouse gases have led to a clear change in the global environmental condition. This global crisis, together with the depletion of fossil resources and the governmental policies toward carbon neutrality, has given rise to an increasing attention toward degradable and/or biobased polymers.^1−6^

One way to make a polymer degradable is to incorporate polar bonds in the polymer backbone, which can break more easily than the C–C bond in typical vinyl (co)polymers. An example is an ester bond that can be quickly hydrolyzed under acidic or alkaline conditions. Nonetheless, incorporating ester bonds into a polymer produced by free-radical polymerization is not an easy task. A very interesting approach to address this challenge is to use cyclic ketene acetals (CKAs) as comonomers.^5,7^ This group of monomers has the ability to polymerize by radical ring-opening polymerization (rROP), creating ester bonds in the main chain (see Figure 1). The radical polymerization gives these monomers the possibility to copolymerize with common vinyl monomers such as styrene, acrylates, or methacrylates while incorporating ester bonds in the main chain. More specifically, a recent review reported that most of the research on copolymerization of CKAs and vinyl monomers has been focused on four different types of vinyl monomers: methacrylates, vinyl acetates, maleimides, and vinyl ethers.^8^

**Figure 1:**
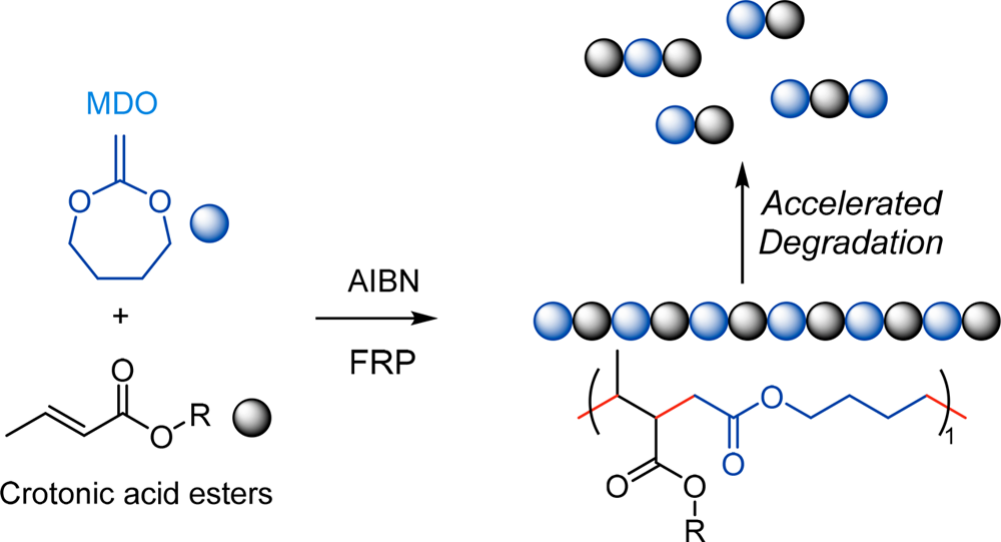
Scheme for the radical copolymerization of crotonate monomers and MDO and subsequent degradation of the alternating copolymer.

The ideal polymerization mechanism is based on a radical addition on the exomethylene group and the subsequent ring-opening process to form an ester group in the main chain. When a radical propagates with the double bond of the CKA, the acetal radical can suffer a β-scission that will open the ring forming the ester group in the polymer backbone (Figure 2a). There is a major challenge though, as there is an undesired competing reaction in the propagation of the acetal radical before β-scission happens, retaining the ring and forming a nondegradable acetal linkage in the polymer backbone (Figure 2a).^9,10^

**Figure 2:**
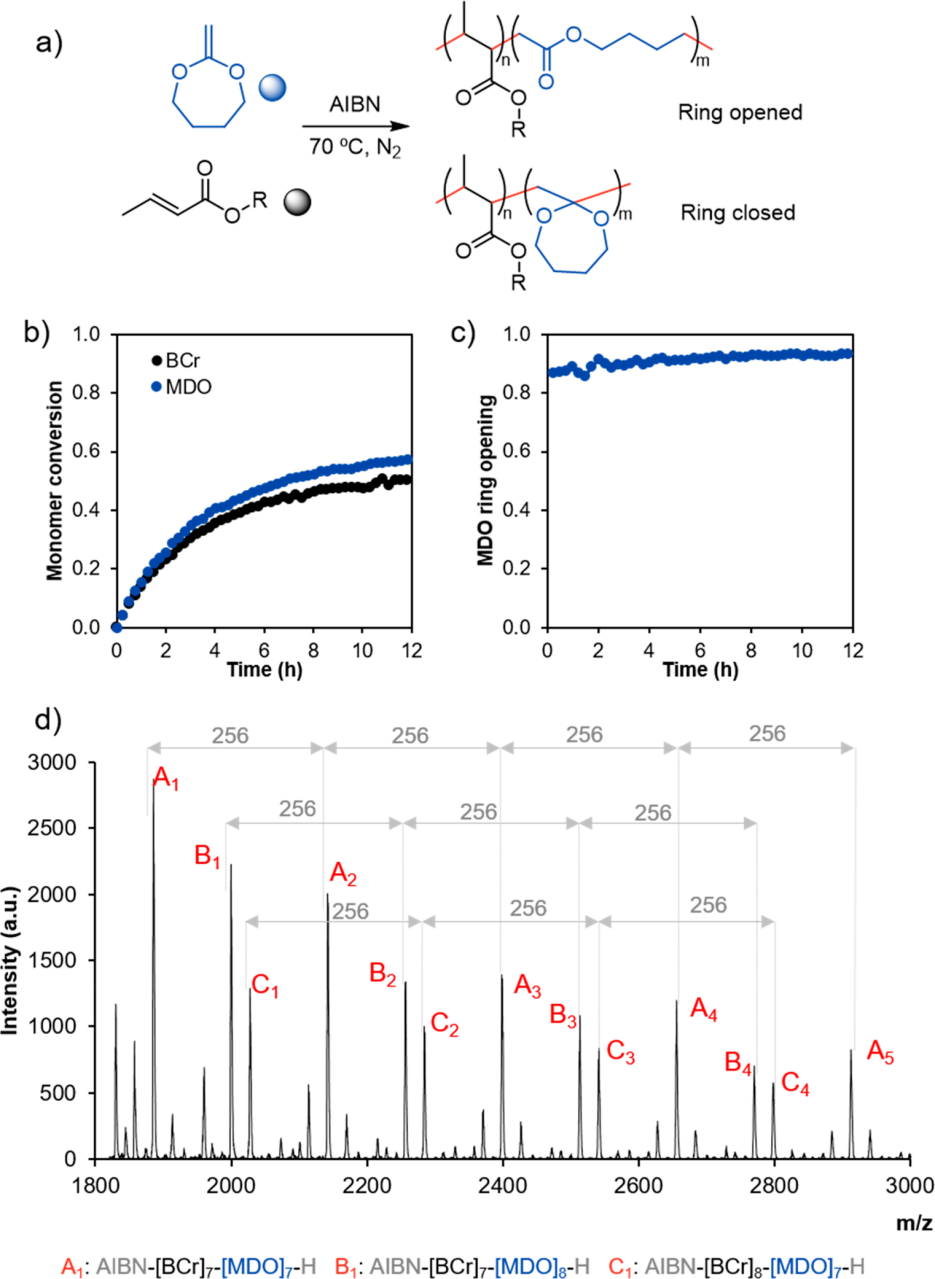
(a) Free radial copolymerization between an alkyl crotonate and MDO, giving ring-opening (up) and ring-retention (down) structures. (b) Time evolution of the individual monomer conversions during the copolymerization of MDO and *n*-butyl crotonate (BCr, when R = (CH_2_)_3_CH_3_) in 50/50 mol ratio. (c) Time evolution of the ring-opening percentage during said reaction. (d) MALDI-TOF spectrum of the copolymer synthesized in bulk.

There is a second key aspect to consider if a good efficiency of the CKAs is desired, and it is that the ester groups must be uniformly distributed along the polymer backbone to ensure uniform degradation of the chain. For this, it is essential to control the copolymer composition and monomer sequence distribution throughout the process. In general, very unfavorable reactivity ratios have been measured for MDO when copolymerized with vinyl acetate, acrylates, or methacrylates, both experimentally^11−14^ and by DFT calculations.^15^ Interestingly there are some exceptions to this trend, as some CKAs form a perfectly alternating copolymer with *N*-alkyl maleimides^16,17^ when copolymerized via RAFT. This is particularly interesting, as the alternating nature of the copolymer ensures a perfect distribution of the ester groups in the copolymer. Thanks to reactivity ratios close to zero for both monomers resulting from radical copolymerization of donor–acceptor (D-A) monomer pairs, alternating copolymers could be obtained. Nevertheless, while decent molecular weights were obtained, in the case of MDO a limited ring-opened amount was obtained (50%) which represented a serious limitation. Moreover, *N*-alkyl maleimides are produced from nonrenewable sources. Very recently, Du et al. found that alternating copolymers can also be produced when CKAs are copolymerized with maleic and itaconic anhydride, provided that these monomers are purified by sublimation.^6^

Inspired by this unique behavior, we set our attention to crotonic acid and derivatives as potential vinyl monomers able to polymerize with MDO. Crotonic acid (CA) is an unsaturated carboxylic acid and structural isomer of methacrylic acid (see Figure 1), with the difference that the methyl group is on the β unsaturated carbon instead of in the α position as in MAA. The great advantage of crotonic acid is that it can be obtained 100% from biomass from the thermal degradation of poly(3-hydroxybutyrate)^18^ in an already industrialized process.^19^ It is important to remark that β-substituted α,β-unsaturated carboxylate monomers, such as alkyl crotonates and alkyl cinnamates, are not easily polymerized.^20,21^ In fact, alkyl crotonates cannot be polymerized by the common radical initiators of azobis(isobutyronitrile) (AIBN) and *rac*-2,2′-azobis(4-methoxy-2,4-dimethylvaleronitrile) (V-70L).

While crotonates have shown a negligible ability to homopolymerize, the free-radical copolymerization of crotonic acid and derivatives with common monomers was demonstrated. Particularly, the crotonic acid–vinyl acetate copolymer was used in many patents, as part of the formulation for products for healthcare,^22^ in the fabrication of textiles,^23,24^ as hot-melt adhesives,^25^ or for ink formulations^26,27^ to name a few. Indeed, the reported low reactivity ratios for crotonates *r*_CA_ ≃ 0 could be beneficial to produce copolymers with good incorporation of MDO.

To determine if the crotonic acid and derivatives could copolymerize efficiently with MDO, we first mixed crotonic acid and MDO in 50 mol % ratio without solvent and monitored the copolymerization by ^1^H NMR. Unfortunately, no polymerization was observed, and all the MDO was hydrolyzed due to the presence of acid functionality (Figure S1, SI). In order to avoid the potential hydrolysis of MDO catalyzed by the crotonic acid, a 100% biobased alkyl crotonate was synthesized through esterification between crotonic acid and 1-butanol following a reported protocol.^28^ (See section 2.5 in the Supporting Information for more details on the monomer synthesis and purification and Figures S4 and S5 for the ^1^H and ^13^C NMR data.) The synthesized monomer (*n*-butyl crotonate, BCr) was copolymerized with MDO in bulk at a 50/50 mol ratio (Figure 2a). The copolymerization was carried out in a NMR tube and *in situ* monitored by ^1^H NMR (Figure S8 in the SI presents a representative ^1^H NMR spectrum taken during the polymerization, and eqs S1–S3 show the calculation of the individual conversions of each monomer and the open percentage of MDO). The polymerization was carried out using the minimum amount of deuterated toluene as solvent (to dissolve the initiator and lock the NMR signal). Figure 2b shows that both monomers react relatively quickly when compared to the individual homopolymerizations. This is particularly surprising in the case of the crotonate monomer, as literature reports indicate that it is unable to homopolymerize. Moreover, both monomers react approximately at the same rate, therefore indicating the formation of a copolymer with a homogeneous composition throughout the copolymerization. Not only that, Figure 2c presents the percentage of ring opening of the MDO during the copolymerization. As observed, more than 90% of the MDO is incorporated as an open ring by the end of the polymerization, producing ester groups in the main chain that can give the polymer the capacity to be degraded. The copolymerization was repeated in bulk in a vial (to obtain a larger amount of material), and the structure of the resulting copolymer was further analyzed by MALDI-TOF, as presented in Figure 2d.

The MALDI-TOF confirms that the copolymer that is formed has an alternating structure. Different populations (noted as A, B, and C in Figure 2d) can be observed, but interestingly, the molar mass between the peaks in each population was 256 Da. The 256 Da molar mass corresponds to the addition of the molar masses of butyl crotonate and MDO (142 and 114 Da, respectively).

A detailed analysis of the identified populations on the MALDI-TOF spectrum is presented in section 3.5.1 of the SI. The most important populations identified share the same ending groups, an initiator fragment, and a H. This suggests that the chains were initiated by the initiator and terminated by chain transfer. The H could also arise from termination by disproportionation, but no evidence of pending double bonds was found in the NMR nor in the MALDI-TOF, and therefore this option was discarded. The main population corresponds to a copolymer with an exact 1/1 ratio of BCr to MDO (population A in Figure 2d). The second and third populations correspond to chains with a 1/1 ratio between the monomers plus an additional unit of MDO or BCr, populations B and C, respectively. Additionally, other populations with a much lower intensity and no chain-ending group were identified. This could fit with chains that were initiated by a monomer radical formed after chain transfer to the monomer and again terminated by chain transfer. This suggests that although the main termination mechanism for the chains is the chain transfer the radicals that are formed do not initiate new chains in high amount. The ratio between the monomers again was 1/1, with an additional BCr unit. These peaks are identified well in Figure S9 of the SI.

The solution copolymerization (in xylene) of BCr and MDO was also carried out in a vial, yielding the properties summarized in Table S1 in the SI. A similar ring-opening percentage was obtained, and similar MALDI-TOF profiles were achieved (see Section 3.5.2. in the SI), confirming the alternating character of the copolymers.

Further, additional copolymerizations were performed again *in situ* in the NMR tube at different monomer ratios (BCr/MDO at 25/75 and 75/25 mol/mol) aiming to estimate the reactivity ratios of the monomer pair. Figure 3a–c shows the time evolution of the individual conversion of each monomer (plus the 50/50 composition already presented in Figure 2b for an easier comparison). Figure 3d shows the time evolution of the cumulative copolymer composition (based on BCr) for each reaction, and Figure 3e shows the evolution of the open MDO percentage.

**Figure 3:**
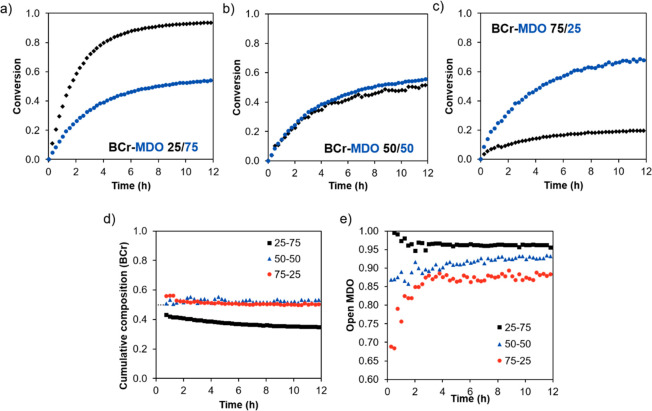
(a–c) Time evolution of the individual conversions of BCr (black) and MDO (blue) during the *in situ* copolymerizations carried out in the NMR tube. (d) Time evolution of the cumulative copolymer composition during reactions a–c. (e) Percentage of open MDO composition during reactions a–c.

When the monomer ratio between MDO and BCr is not equal (Figure 3a and Figure 3c), the monomer conversion plots do not overlap. In both cases, the monomer in the lowest amount has a higher conversion through all of the experiment. This could initially lead to the belief that the monomers are not reacting at the same rate. However, this is not the case, as it is important to note that the rate of conversion (or in other words, the slope of the plots presented in Figure 3a–c) is not the same as the rate of reaction. The rate of reaction is the product of the rate of conversion × the initial monomer concentration. Thus, taking the experiment in Figure 3c as an example, the rate of conversion (slope) of MDO is 3 times higher than that of BCr, but as the initial monomer concentration of MDO is 3 times lower, the rate of polymerization of both monomers results in the same value. The result of this is that the evolution of the cumulative copolymer composition (Figure 3d, red) is constant at 0.5.

Figure 3d also shows that when the concentration of MDO is higher the behavior is not purely alternating, as the copolymer composition is below 50% BCr in the entire interval. This is likely because the MDO monomer is capable of homopropagation, and as a result, occasionally additional MDO units are incorporated in the copolymer chains. On the contrary, BCr is incapable of homopropagation. As a result, when BCr is in high concentration, no additional BCr is added to the copolymer, and only the alternating copolymer is produced.

The percentage of open MDO (Figure 3e) shows that as the BCr/MDO ratio increases the amount of open MDO slightly decreases, although it is higher than 85% by the end of the reaction in all cases.

The MALDI-TOF spectra of the samples are presented in Figure 4. In both cases, the same 256 Da repeating pattern that was observed for the 50/50 composition (Figure 2d) can be identified. The spectrum of the 75/25 BCr/MDO composition (Figure 4b) is very similar to that of the 50/50 one, although the relative intensities of the peaks are different. Here, those populations with one or two additional BCr units are more intense, while the populations with additional MDO units are not present; however, the overall composition of the chains is very close to 50% of each monomer. The spectrum of the 25/75 BCr/MDO copolymer is quite complex, on the other hand. Although the same 256 Da repeating pattern can be observed, the number of peaks is much larger. The additional peaks correspond to chains where the MDO fraction is larger than 50%, which is in agreement with what was observed in the evolution of the copolymer composition in Figure 3d. A detailed identification of the most relevant peaks of each spectrum is presented in sections 3.5.3 and 3.5.4 of the Supporting Information.

**Figure 4:**
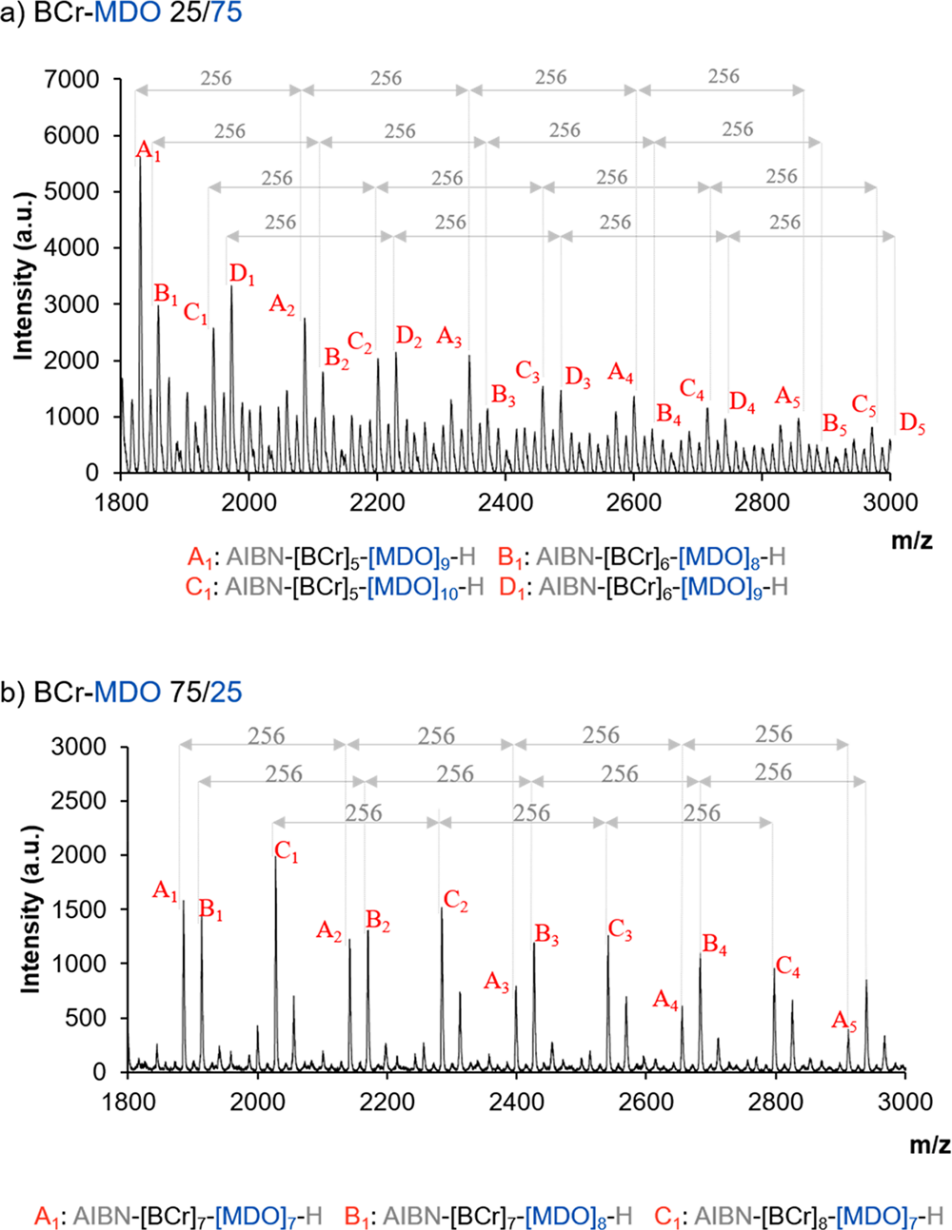
MALDI-TOF spectra of BCr/MDO copolymers produced in a 25/75 and 75/25 mol ratio.

A nonlinear least squares method (NLLSQ) based on the Mayo–Lewis composition equation developed by De la Cal et al.^29^ was used to estimate the reactivity ratios by fitting the evolution of the cumulative copolymer composition as a function of overall monomer conversion for the copolymerizations carried out at different BCr/MDO ratios. The reactivity ratio values were estimated as *r*_BCr_ = 0.017 ± 0.007 and *r*_MDO_ = 0.105 ± 0.013. The comparison of the predicted and experimental evolution of the cumulative copolymer composition over the total conversion is presented in Figure S14 in the SI. As expected, both reactivity ratios are well below one, particularly for butyl crotonate, showing a preference to react with the other monomer and thus produce an alternating copolymer. Using the estimated reactivity ratios, the instantaneous composition of the copolymer (*F*_BCr_) was plotted over the monomer feed composition (*f*_BCr_), using the Mayo–Lewis equation^30^ (see Figure S15 in the SI). The plot clearly shows that for a large range of monomer compositions (*f*_BCr_ = 45–80%) the obtained instantaneous copolymer composition is nearly independent of the monomer feed composition, and *F*_BCr_ ∼ 50% is obtained. Thus, the copolymerizations in 50/50 and 75/25 ratios, which fall into this region, produce copolymers with 50% composition and alternating structure. On the contrary, the copolymerization with 25/75 monomer ratio is not in that region. Consequently, the copolymer composition is lower than 50% (Figure 3c), and the MALDI-TOF spectrum (Figure 4a) is more complex.

The degradability of the BCr-MDO copolymer was tested by adapting a protocol described elsewhere.^31^ In short, 100 mg of polymer was dissolved in 8 mL of THF. Next, a solution of 240 mg of KOH in 2.5 mL of MeOH was added, and the reaction mixture was stirred at room temperature. At different reaction times, 1 mL aliquots were taken and quenched with 50 μL of an aqueous solution of HCl (6 M). One mL of THF was added to the resulting suspension and filtered through a PTFE filter (0.45 μm). After rotary evaporation of the solvent, the samples were dried under a vacuum for 16 h. The resulting samples were redissolved in 2 mL of chloroform, filtered, and injected for SEC/RI equipment. The evolution of the molar mass is presented in Figure 5.

**Figure 5:**
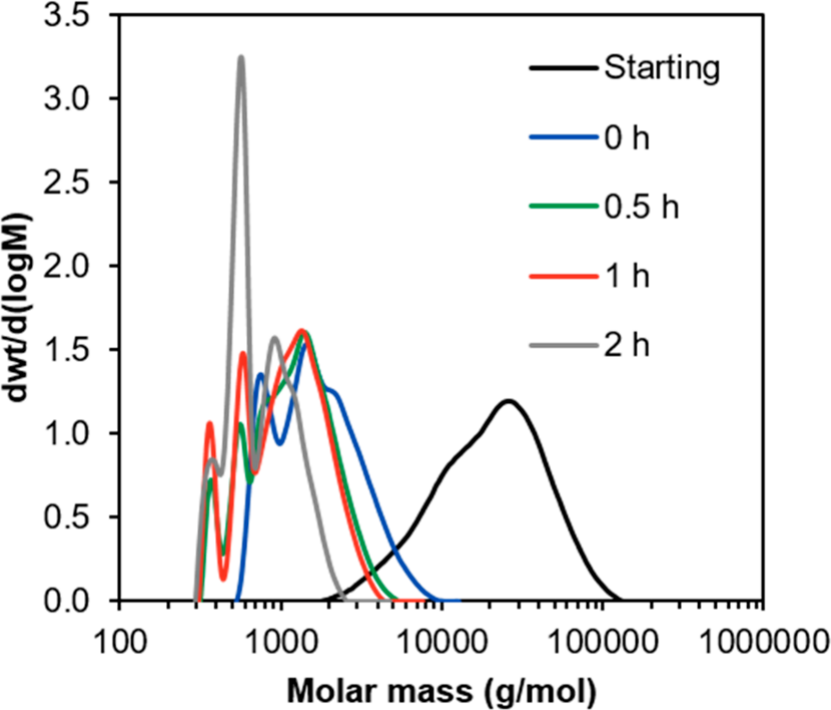
Evolution of the molar mass distribution of the BCr-MDO copolymer under the degradation conditions.

Figure 5 shows the time evolution of the molar mass during the degradation experiments. The sample labeled as “starting” corresponds to the sample before the degradation experiment, while the one labeled “0 h” corresponds to the sample after it was mixed with the MeOH/KOH solution but neutralized immediately (in less than 10 s). There is a clear difference between these two samples as the molar mass of the “0 h” sample has a much lower molar mass. This is likely because some of the KOH was not completely neutralized, so the sample continued to degrade (and therefore reduce the molar mass) during the filtering and drying steps. The samples at higher degradation times show even lower molar masses, showing that the copolymer continued to degrade over this time. The extremely fast degradation of the polymer must be a consequence of the alternating structure of the copolymer, which allows a perfect distribution of the degradable moieties over the chain. This can also be corroborated by the small molar mass of the fragments (below 2 kg/mol). An ^1^H NMR study of the degradation products, showing evidence of the hydrolysis of the ester groups in the backbone ester, is presented in Figures S24 and S25.

The work has been expanded to other crotonate monomers, namely, ethyl crotonate and 2-octyl crotonate. Table 1 shows a summary of the main properties of these copolymers (50% crotonate, 50% MDO), compared to those of butyl crotonate, all of them copolymerized in bulk at 75 °C. The polymerization kinetics and time evolution of the open MDO are presented in Figures S16 and S17 in the SI. Figures S18 and S19 show the ^1^H NMR spectra of these polymerizations at time 0 and 12 h after polymerization with the identification of the monomer and polymer signals.

**Table 1:** Properties of the Bulk Copolymerization of Different Alkyl Crotonates with MDO

Crotonate monomer	Conversion (in 6 h)^a^	*T*_g_ (°C)^b^	Ring open (%)^c^
Ethyl crotonate	80.5	–29	95
Butyl crotonate	85.0	–37	93
2-Octyl crotonate	82.5	–41	97

a Individual monomer conversions were monitored by *in situ*^1^H NMR and presented in Figures S16, S18, and S19.

b DSC traces of the three copolymers are shown in Figure S22.

c See Figure S17 for the time evolution of the cumulative ring opening during the polymerization reaction.

As observed, very similar properties were obtained for all crotonates. Both ECr-MDO and 2OCr-MDO copolymers showed the same alternating behavior in their corresponding MALDI-TOF spectrum (Figures S20 and 21, respectively) and degraded similarly too (Figures S23 and S24). Thus, we can conclude that the interesting copolymerization behavior of *n*-butyl crotonate with MDO can be extended to other alkyl crotonates. Furthermore, changing the ester group allowed tuning the *T*_g_ of the final copolymer, paving the way to the synthesis of degradable and biobased copolymers with a wide range of *T*_g_s by using other bulkier alcohols in the synthesis of the crotonates.

In conclusion, in this work, we have shown novel degradable and biobased polymers produced by the free-radical copolymerization between a cyclic ketene acetal (2-methylen-1,3-dioxepane, MDO) and ester derivatives of the crotonic acid. Both monomers are interesting on their own but have hardly been used because their copolymerization behavior with common monomers such as styrene, acrylates, or methacrylates are very unfavorable. In the case of the crotonates, in addition to issues regarding the reactivity ratios, extremely low propagation rate coefficients make them nearly unusable. Nonetheless, the combination of MDO and butyl crotonate presented surprisingly efficient copolymerization behavior. The MALDI-TOF analysis of the copolymer revealed an alternating copolymer as the repeating unit was that of the MDO-BCr dimer. This was supported by the calculated reactivity ratios, which were well below the unit, leading to the alternating behavior in a range of feed compositions between 45 and 80% of BCr. The scope of these copolymers was then expanded by showing that the same behavior is observed for other crotonate monomers, further widening the possible range of applications.

## Supporting Information Available

Additional experimental details including materials, methods, NMR, and MALDI-TOF spectra, equations, additional copolymerization kinetics, DSC plots, and degradation experiments (PDF)
