# Supplementary material for: Degradable Alternating Copolymers by Radical Copolymerization of 2-Methylen-1,3-dioxepane and Crotonate Esters
Source: ACS Macro Lett. 2024 Mar 8;13(3):368–74. doi: 10.1021/acsmacrolett.4c00101 (PMC10956490; doi:10.1021/acsmacrolett.4c00101)
Supplement: Supplementary file 1 — mz4c00101_si_001.pdf [file mz4c00101_si_001.pdf]

Supporting information for:

Degradable alternating copolymers by radical copolymerization of 2-methylen-1,3-dioxepane and crotonate esters

Aitor Barquero[1], Arianna Zanoni[2], Elena Gabirondo[3], Estibaliz González de San Román[4], Shaghayegh Hamzehlou[1], Marta Ximenis[3], Davide Moscatelli[2], Haritz Sardon[3], Jose Ramon Leiza[1*]*

1-POLYMAT and Department of Applied Chemistry, University of the Basque Country UPV/EHU, Joxe Mari Korta Center, Tolosa hiribidea, 72, 20018 Donostia, Spain

2-Department of Chemistry, Materials and Chemical Engineering “Giulio Natta”, Politecnico di Milano, via Mancinelli 7, 2013 Milano, Italy

3-POLYMAT and Department of Polymers and Advanced Materials: Physics, Chemistry and Technology, Faculty of Chemistry, University of the Basque Country UPV/EHU, Paseo Manuel de Lardizabal 3, 20018 Donostia-San Sebastián, Spain

4-POLYMAT, University of the Basque Country UPV/EHU, Joxe Mari Korta Center, Tolosa hiribidea, 72, 20018 Donostia, Spain

Corresponding authors: Haritz Sardon (haritz.sardon@ehu.eus) and Jose Ramon Leiza (jrleiza@ehu.eus).

1. Materials 2
2. Methods 2
2.1. Nuclear Magnetic Resonance (NMR) 2
2.2. Matrix-Assisted Laser Desorption/Ionization- Time Of Flight (MALDI-TOF) 2
2.3. Size Exclusion Chromatography (SEC) 2
2.4. Differential Scanning Calorimetry (DSC) 3
2.5. Synthesis of the crotonate monomers 3
2.6. Copolymerization of alkyl crotonates with MDO in bulk 3
3. Results 4
3.1. Copolymerization of crotonic acid and MDO 4
3.2. Characterization of the biobased crotonate monomers 4
3.2.1. Ethyl crotonate (ECr) 4
3.2.2. n-Butyl crotonate (BCr) 5
3.2.3. 2-octyl crotonate (2OCr) 6
3.3. Copolymerization of BCr and MDO 7
3.4. Properties of the BCr-MDO copolymer synthetized by solution polymerization 9
3.5. Detailed identification of the MALDI-TOF spectra of the BCr-MDO copolymers 9
3.5.1. BCr-MDO 50/50 by bulk copolymerization 9
3.5.2. BCr-MDO 50/50 by solution copolymerization in xylene 10
3.5.3. BCr-MDO 25/75 by bulk copolymerization 11
3.5.4. BCr-MDO 75/25 by bulk copolymerization 12

3.6. Fitting of the reactivity ratios and Mayo-Lewis equation 12
3.7. Copolymerization of ECr and 2OCr with MDO 13
3.7.1. Copolymerization kinetics 13
3.7.2. Molar mass distribution of ECr and 2OCr copolymers......16
3.7.3. MALDI-TOF spectra of the ECr and 2OCr copolymers 16
3.7.4. DSC traces of crotonate-MDO copolymers 17
3.7.5. Degradation experiments of the ECr and 2OCr copolymers 18

1. Materials

Ethanol (EtOH, 99.8%, Sigma-Aldrich), butanol (BuOH, 99.9%, Sigma-Aldrich), 2-octanol (2-OcOH, 97%, Sigma-Aldrich), sulfuric acid (H_2SO_4, 95-98%, Sigma-Aldrich), crotonic acid (CA, Sigma-Aldrich), chloroform ($CHCl_3$, Fisher), sodium bicarbonate ($NaHCO_3$, Sigma-Aldrich), 2,2'-azobisisobutyronitrile (AIBN, Aldrich), xylene (Sigma-Aldrich) and deuterated chloroform ($CDCl_3$, Eurisotop) were used as received. 2-methylen-1,3 dioxepane (MDO) was kindly supplied by Waker Chemie. HPLC grade chloroform ($CHCl_3$, Sharlau) was used for the molar mass measurements.

2. Methods

2.1. Nuclear Magnetic Resonance (NMR)

The spectra were obtained in a Bruker Advance DPX 300, using deuterated chloroform ($CDCl_3$) as solvent at room temperature. For the ^{1}H-NMR measurement 10 mg of sample was used, 3 s of acquisition time, 1 s delay time, 8.5 µs pulse and a spectral width of 5000 Hz and 32 scans. In the case of ^{13}C-NMR 40 mg of sample, 3 s of acquisition time, 4 s of delay time, 5.5 µs pulse and 18800 Hz spectra width and 10000 scans were employed.

2.2. Matrix-Assisted Laser Desorption/Ionization- Time Of Flight (MALDI-TOF)

MALDI-TOF MS measurements were performed on a Bruker Autoflex Speed system (Bruker, Germany) equipped with a Smartbeam-II laser (Nd:YAG, 355 nm, 2 kHz). Spectra were acquired in reflectron mode; each mass spectrum was the average of 10000 shots. The laser power was adjusted during the experiments. Polymer samples were dissolved in THF at a concentration of 10 mg·mL^{-1}. DCTB was used as a matrix. The matrix was dissolved in THF at a concentration of 10 mg·mL^{-1}. NaTFA was used as cation donor (10 mg·mL^{-1} dissolved in THF). The polymer samples were mixed with the matrix and salt at a 10:3:1 (matrix/polymer/salt) ratio. Approximately 0.5 µL of the obtained solution was spotted by hand on the ground steel target plate and allowed to dry in air. Spectra were accumulated and processed using FlexControl (v3.4) and FlexAnalysis softwares (v3.4), respectively. Peaks were detected in SNAP mode with a signal-to-noise threshold of 3.00 before being processed with a Savitzky–Golay smoothing algorithm (0.05 m·z^{-1} width, one cycle) and "TopHat" baseline sub-traction. External calibration was performed in quadratic mode with a mixture of different polystyrene standards (PS, Varian).

2.3. Size Exclusion Chromatography (SEC)

The samples were first dried in an oven at 60 °C under vacuum and then redissolved in $CHCl_3$ to achieve a concentration of about 1 mg·mL^{-1}. The solutions were filtered with a PTFE filter (pore size = 0.45 µm) before

they were injected into the SEC instrument. The SEC instrument was an “Agilent PL-GPC 50 integrated system” consisting on an autosampler, a pump and a differential refractometer as detector. A guard column (Agilent PLGel 5µM, 50x1.5mm and two columns (PLGel µM, 30x7.5mm) were used for the fractionation at 40 °C using $CHCl_3$ with 0.5% of triethylamine as eluent. The flow rate of $CHCl_3$ through the columns was 1 $mL·min^{-1}$. The reported molar masses are referred to polystyrene standards and the data was processed with PL-GPC software control v.2.3.0 (Agilent). .

2.4. Differential Scanning Calorimetry (DSC)

The crystallinity measurements were performed using a DSC8500 from Perkin Elmer, Inc. calibrated with indium and tin standards. The DSC scans were performed with approximately 5 mg of film sample to calculate the glass transition temperature. In all cases the first scan was performed to eliminate the thermal history of the samples and after that the samples were analyzed between -60 ºC 150 ºC in a heating rate of 20 ºC/min. The glass transition temperatures were calculated from the second heating run.

2.5. Synthesis of the crotonate monomers

Bio-based crotonate monomers were synthetized by Fisher esterification of the crotonic acid with alkyl alcohols of different lengths. The alcohols (ethanol, n-butanol and 2-octanol) were selected among the alcohols that can be fully obtained from renewable sources. The synthesis of n-butyl crotonate (BCr) will be explained in detail as example.

The esterification was carried out in a 250 mL round bottom flask. 10 g of crotonic acid (0.12 mol) and 10.33 g of butanol (0.14 mol) were dissolved in chloroform (150 mL) and 1 mL of H_2SO_4 was added as a catalyst. The flask was left stirring under reflux at 65 °C overnight.

The product was washed twice with a separation funnel with $NaHCO_3$ (1M) solution to get rid of sulfuric acid, unreacted alcohol and crotonic acid. The chloroform solution was then dried with a rotary evaporator and the product was collected in a vial.

2.6. Copolymerization of alkyl crotonates with MDO in bulk

Crotonic ester derivatives were polymerized with 2-methylen-1,3-dioxepane in bulk and solution at different molar ratios (25/75, 50/50 or 75/25). The reactions were carried out under controlled temperature using a heating plate and a disk to place the vials. The different copolymers were synthesized using 2 wt% of AIBN as initiator and different butyl crotonate/2-methylen-1,3-dioxepane (BCr/MDO) ratios: 25/75 (0.293 g, 2.06 10^{-3} mol/ 0.707 g, 6.19 10^{-3} mol) 50/50 (0,554 g, 3.90 10^{-3} mol/ 0,445g, 3.90 10^{-3} mol) 75/25 (0.789 g, 5.55 10^{-3} mol/ 0.211 g, 1.85 10^{-3} mol). The reaction were purged with nitrogen and then left at 75 °C for 6 hours while stirring. Every hour a small aliquot was extracted to calculate the conversion of each monomer by ^{1}H-NMR. The solution copolymerizations were carried out using xylene as solvent at 70 % solids content.

3. Results

3.1. Copolymerization of crotonic acid and MDO

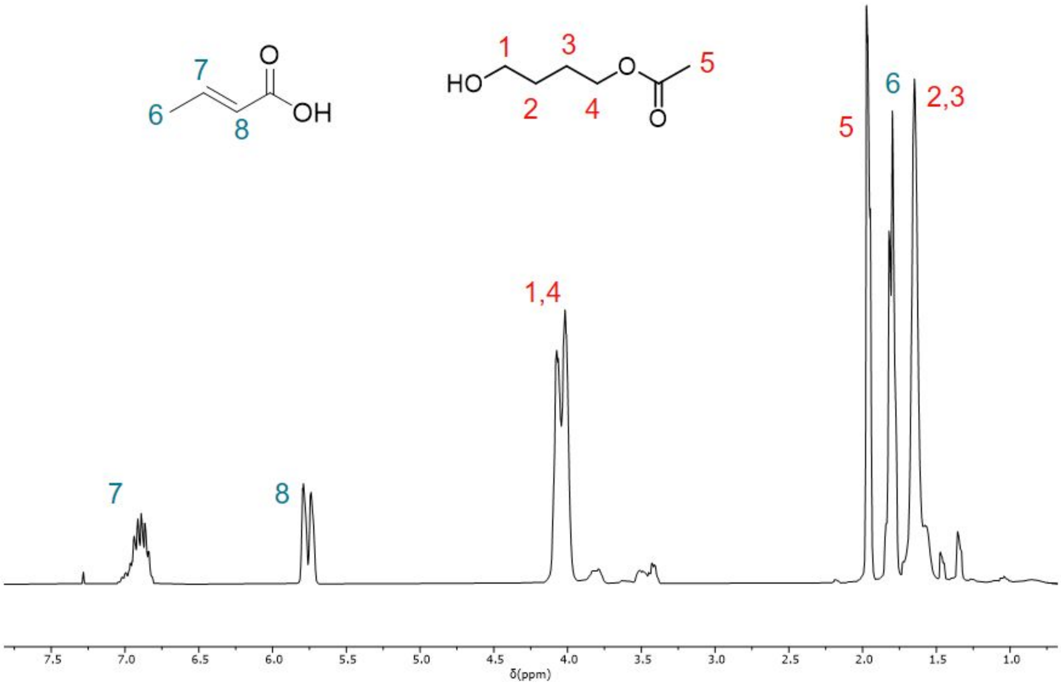

Figure S1. ^{1}H-NMR spectrum of the reaction between crotonic acid and MDO, where the complete hydrolisation of MDO could be observed. The spectrum shows the peaks (1 to 5) corresponding to the 4-hydroxy-1-butylacetate, which is the hydrolysis product, and peaks (6-8) that correspond to the unreacted crotonic acid.

3.2. Characterization of the biobased crotonate monomers

In this section the ^{1}H and ^{13}C-NMR of the synthetized alkyl crotonate monomers is presented.

3.2.1. Ethyl crotonate (ECr)

Figures S2 and S3 present the ^{1}H and ^{13}C-NMR of ethyl crotonate (ECr) monomer, respectively.

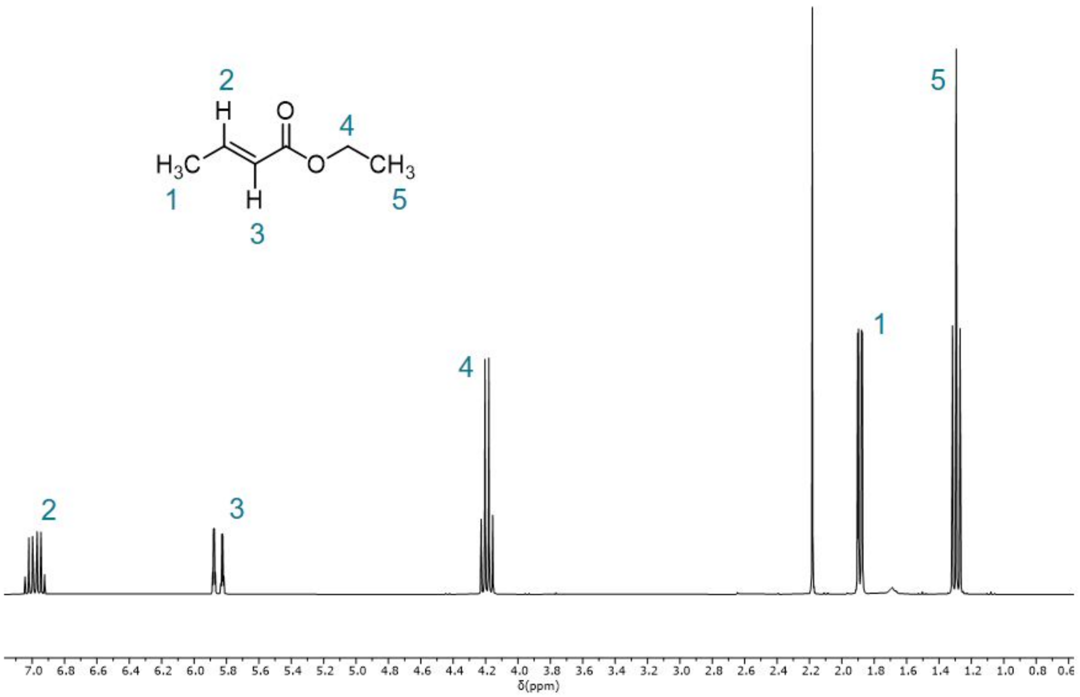

Figure S2. *^{1}H-NMR spectrum of ethyl crotonate (ECr).*

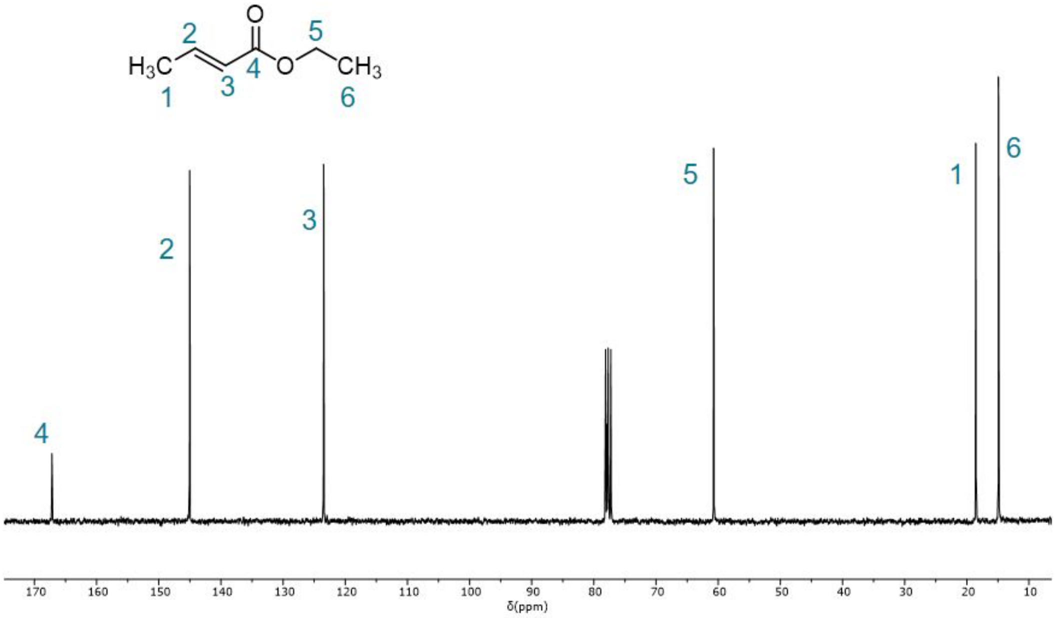

Figure S3. *^{13}C-NMR spectrum of ethyl crotonate (ECr).*

3.2.2. n-Butyl crotonate (BCr)

Figures S4 and S5 present the ^{1}H and ^{13}C-NMR of n-butyl crotonate (BCr) monomer, respectively.

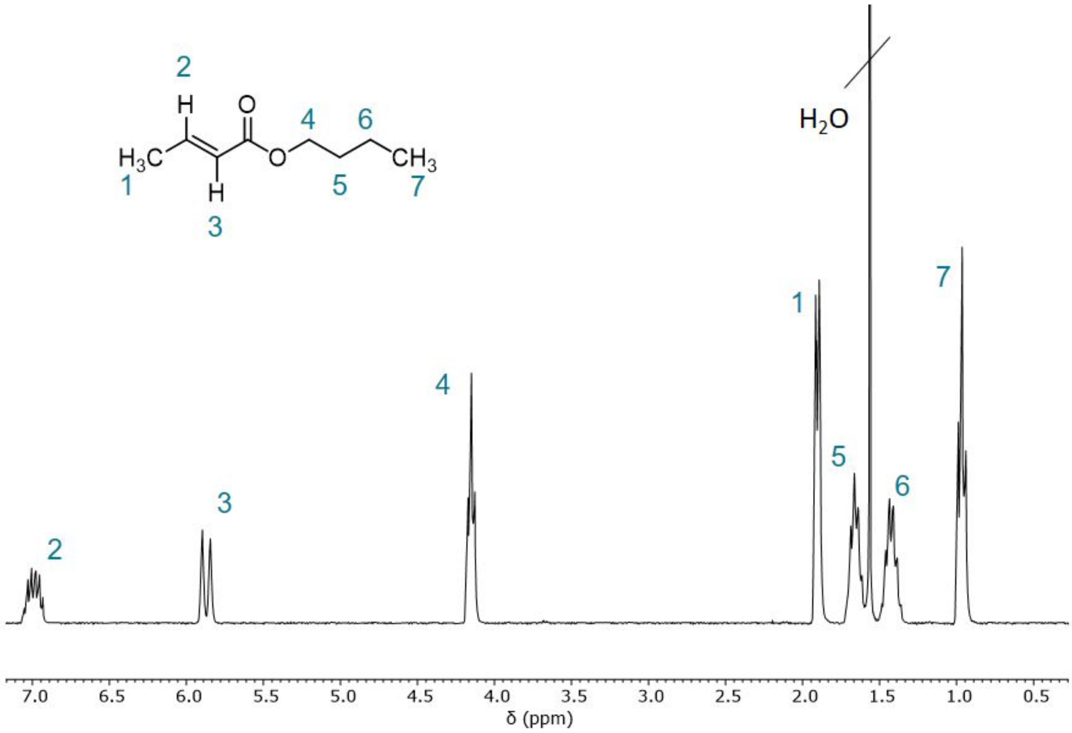

Figure S4. *^{1}H-NMR spectrum of butyl crotonate (BCr).*

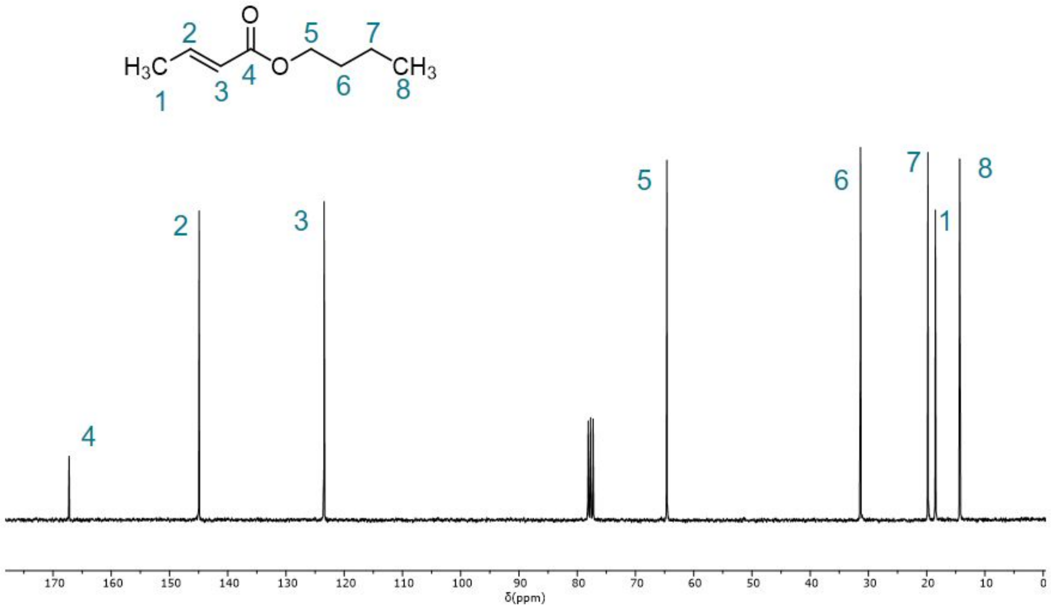

Figure S5. *^{13}C-NMR spectrum of butyl crotonate (BCr).*

3.2.3. 2-octyl crotonate (2OCr)

Figures S6 and S7 present the ^{1}H and ^{13}C-NMR of 2-octyl crotonate monomer (2OCr), respectively.

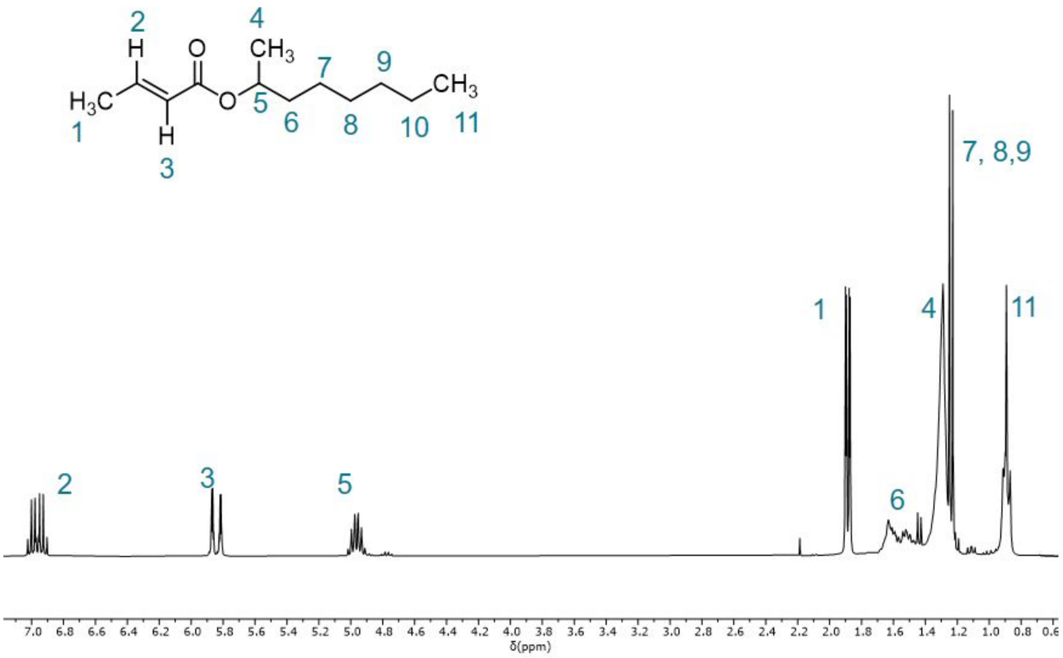

Figure S6. *^{1}H-NMR spectrum of 2-octyl crotonate (2OCr).*

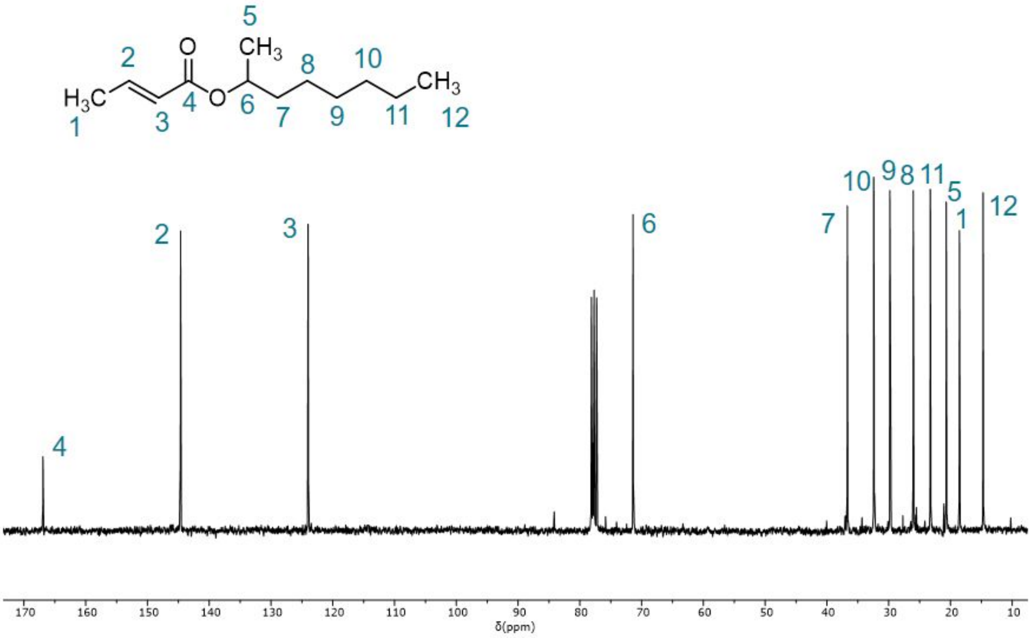

Figure S7. *^{13}C-NMR spectrum of 2-octyl crotonate (2OCr).*

3.3. Copolymerization of BCr and MDO

Figure S8 shows the ^{1}H-NMR of the copolymerization between BCr and MDO at the beginning of the reaction and after 12 h of reaction time with the assignation of the signals, both in the monomers and in the copolymer.

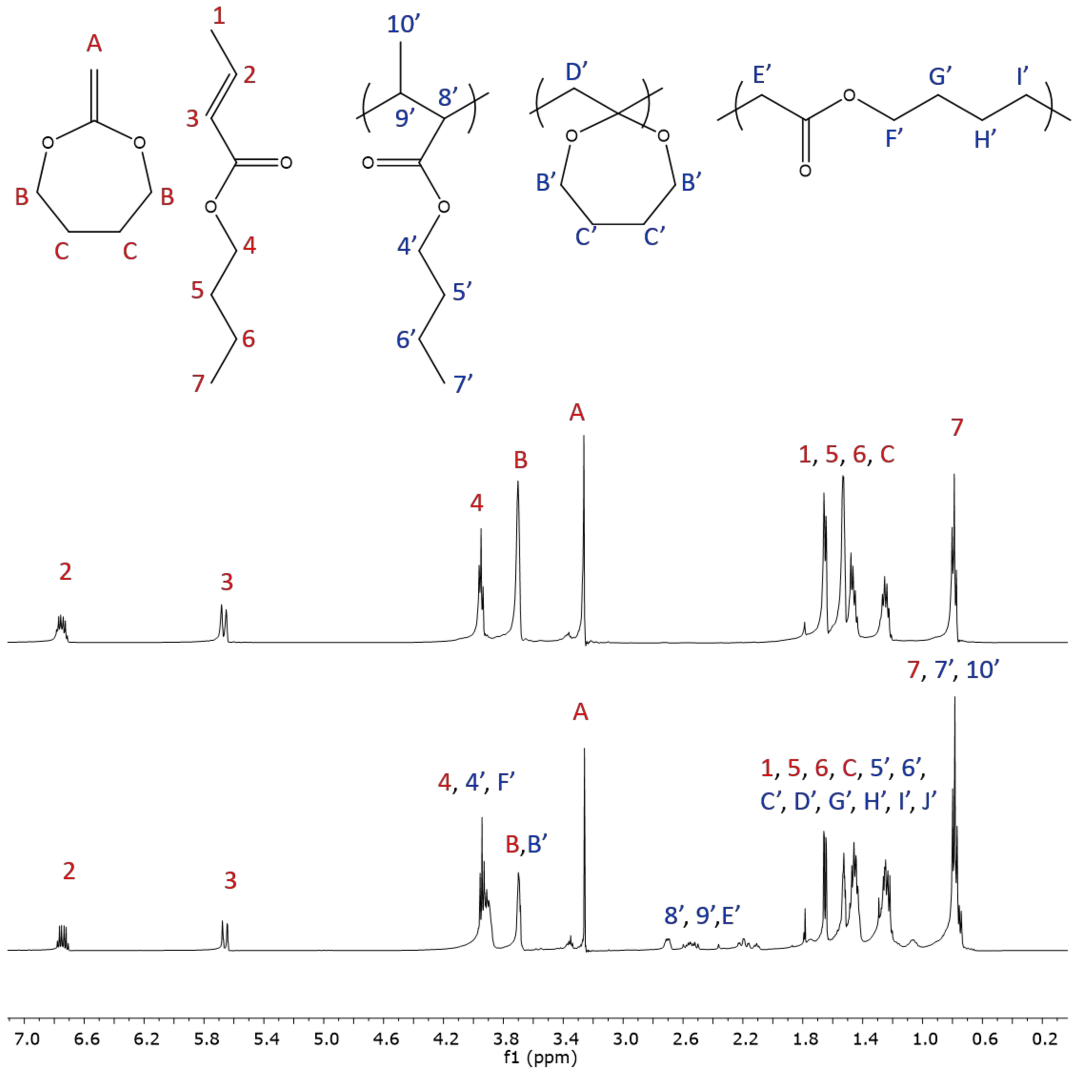

Figure S8. *^{1}H-NMR of the copolymerization between BCr and MDO in a 50-50 mol ratio at the beginning of the reaction (top) and after 12 h (bottom).*

The individual monomer conversions were calculated at any time by directly integrating the signals corresponding to the double bonds and comparing them to the initial area. The open percentage of MDO was calculated comparing the disappearance of the acetal signals (B and B’) with respect to the disappearance of the double bond (A).

$$x_{BCr} = 1 - \frac{A_2}{A_{2,0}} \qquad \text{(eq. S1)}$$

$$x_{MDO} = 1 - \frac{A_A}{A_{A,0}} \qquad \text{(eq. S2)}$$

$$MDO_{open}\,(\%) = \frac{1 - \frac{A_{B,B'}}{A_{B,0}}}{1 - \frac{A_A}{A_{A,0}}} * 100 \qquad \text{(eq. S3)}$$

where x_{BCr} and x_{MDO} are the conversions of BCr and MDO, A_2, A_A, $A_{B,B'}$ are the areas of peaks 2, A and B,B’ at time t, and $A_{2,0}$, $A_{A,0}$, $A_{B,0}$ are the areas of peaks 2, A and B, at time 0.

The cumulative copolymer composition was calculated from the conversions and initial monomer concentrations, using equation S4.

$$Y_{BCr} = \frac{x_{BCr}[BCr]_0}{x_{BCr}[BCr]_0 + x_{MDO}[MDO]_0} \qquad \text{(eq. S4)}$$

where x_{BCr} and x_{MDO} are the conversions of the crotonate and MDO, respectively, and $[BCr]_0$ and $[MDO]_0$ are the initial concentrations of the butyl crotonate and MDO, respectively.

3.4. Properties of the BCr-MDO copolymer synthetized by solution polymerization

A summary of the most relevant properties of the copolymer can be found in Table S1

Table S1. Summary of the properties of the BCr-MDO copolymer synthetized by solution polymerization in xylene.

x_{BCr}	x_{MDO}	Ring open (%)	M_n (g/mol)	M_w (g/mol)
0,78	0,62	73,2	2803	5515

3.5. Detailed identification of the MALDI-TOF spectra of the BCr-MDO copolymers

3.5.1. BCr-MDO 50/50 by bulk copolymerization

Figure S9 presents a zoom of the MALDI-TOF spectrum presented in Figure 2d. The structures identified in the spectrum are summarized in Table S1.

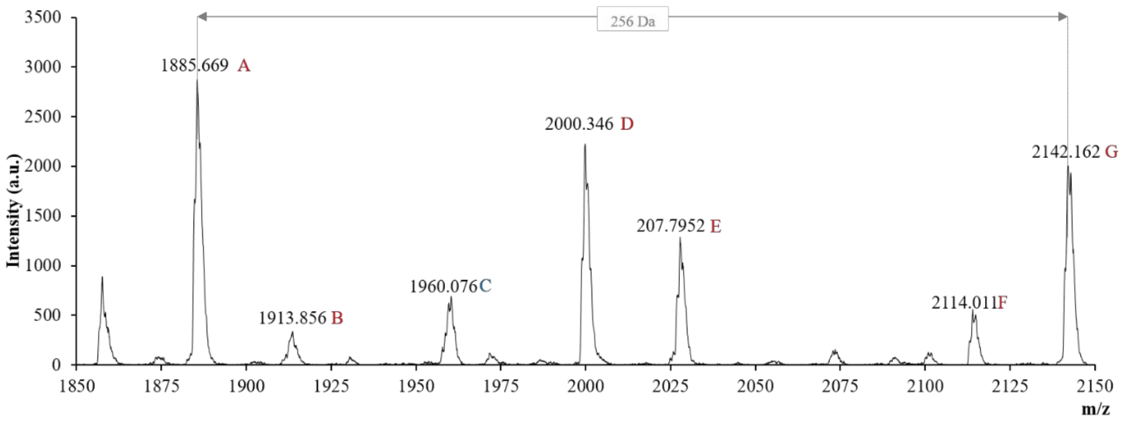

Figure S9. *Zoom of the MALDI-TOF spectrum of BCr-MDO 50/50 copolymer presented in Figure 2d for the identification of the peaks.*

Table S2. *Identification of the peaks from Figure S9.*

Peak	Structure
A	AIBN-$[BCr]_7$-$[MDO]_7$-H
B	AIBN-$[BCr]_8$-$[MDO]_6$-H
C	$[BCr]_8$-$[MDO]_7$
D	AIBN-$[BCr]_7$-$[MDO]_8$-H

E	AIBN-$[BCr]_8$-$[MDO]_7$-H
F	AIBN-$[BCr]_7$-$[MDO]_9$-H
G	AIBN-$[BCr]_8$-$[MDO]_8$-H

3.5.2. BCr-MDO 50/50 by solution copolymerization in xylene

Figure S10 shows the MALDI-TOF spectrum of the BCr-MDO copolymer synthesized by solution polymerization in xylene. The same pattern is observed every 256 Da, similarly to what was presented in Figure 2d for the bulk copolymer. Figure S11 shows a zoom of the same spectrum, for the identification of each population (In table S3).

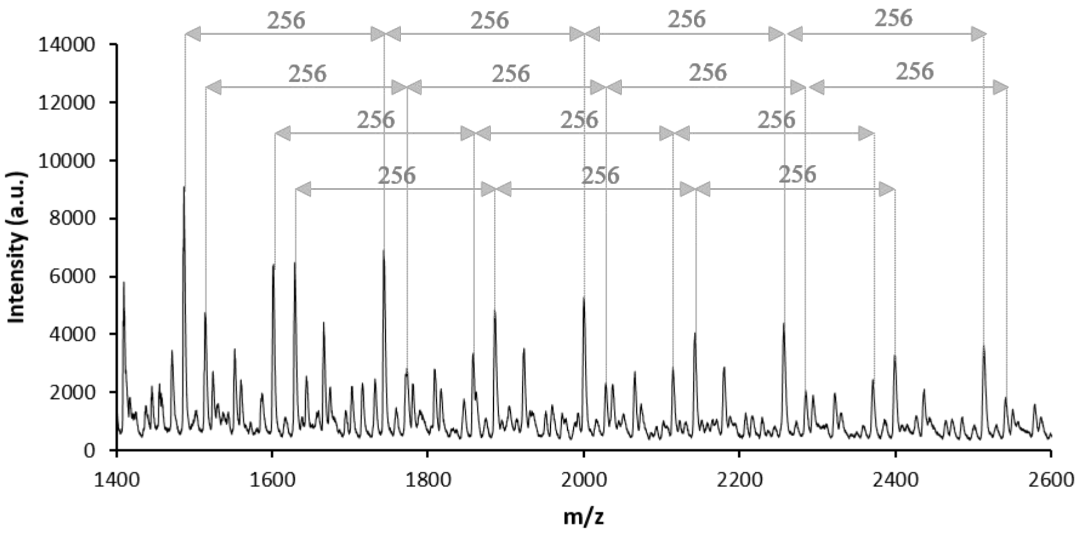

Figure S10. *MALDI-TOF spectrum of the BCr-MDO copolymer synthesized by solution polymerization in xylene, showing the alternating nature by the repetition of the 256 Da repeating unit.*

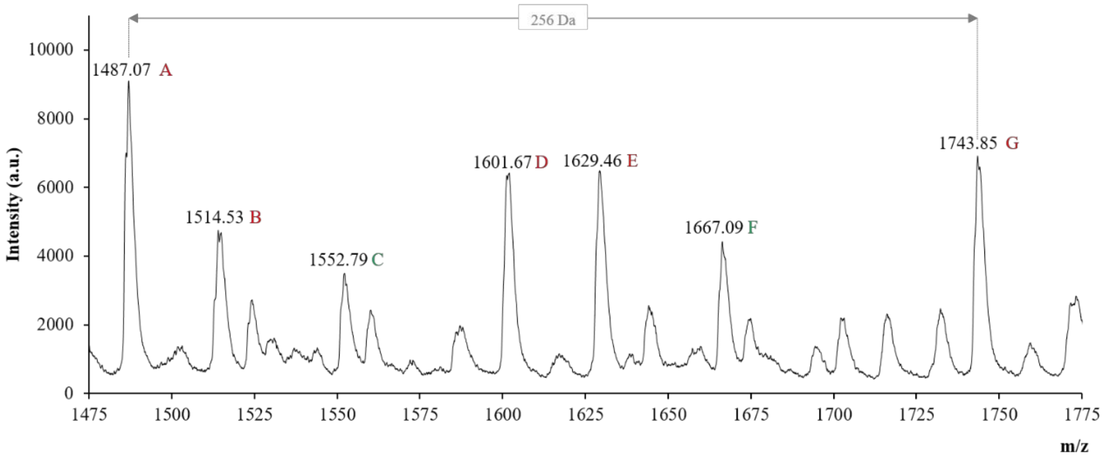

Figure S11*. Zoom of the MALDI-TOF spectrum of the BCr-MDO copolymer synthesized by solution polymerization in xylene for the identification of the peaks.*

Table S3*. Identification of the peaks from Figure S11.*

Peak	Structure
A	AIBN-$[BCr]_5$-$[MDO]_6$-H
B	AIBN-$[BCr]_6$-$[MDO]_5$-H

C	Xylene-$[BCr]_6$-$[MDO]_5$-H
D	AIBN-$[BCr]_5$-$[MDO]_7$-H
E	AIBN-$[BCr]_6$-$[MDO]_6$-H
F	Xylene-$[BCr]_6$-$[MDO]_6$-H
G	AIBN-$[BCr]_6$-$[MDO]_7$-H

3.5.3. BCr-MDO 25/75 by bulk copolymerization

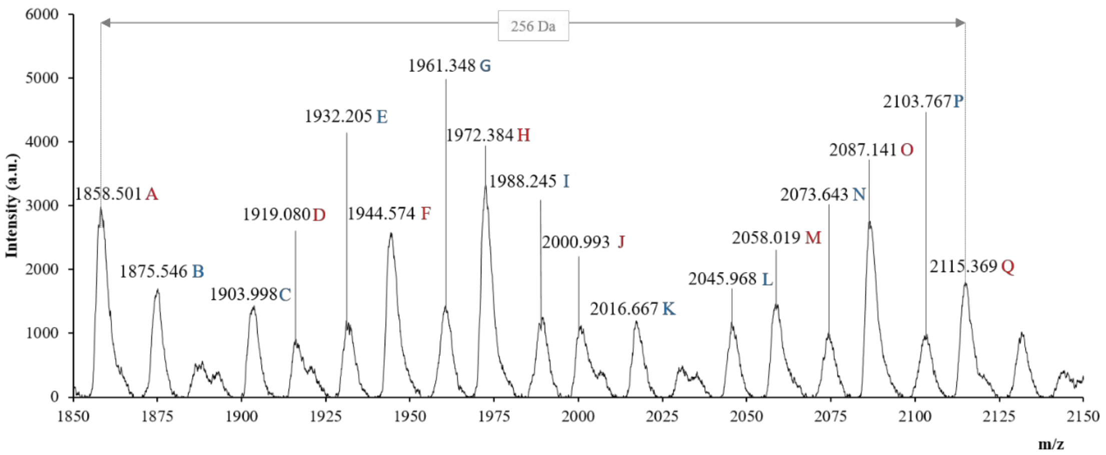

***Figure S12**. Zoom of the MALDI top of the BCr-MDO 25/75 copolymer, presented in Figure 4a, for the identification of the peaks*

***Table S4**. Identification of the peaks from Figure S12.*

Peak	Structure
A	AIBN-$[BCr]_6$-$[MDO]_8$-H
B	$[BCr]_5$-$[MDO]_{10}$
C	$[BCr]_6$-$[MDO]_9$
D	AIBN-$[BCr]_4$-$[MDO]_{11}$-H
E	$[BCr]_7$-$[MDO]_8$
F	AIBN-$[BCr]_5$-$[MDO]_{10}$-H
G	$[BCr]_8$-$[MDO]_7$
H	AIBN-$[BCr]_6$-$[MDO]_9$-H
I	$[BCr]_5$-$[MDO]_{11}$
J	AIBN-$[BCr]_7$-$[MDO]_8$-H
K	$[BCr]_6$-$[MDO]_{10}$
L	$[BCr]_7$-$[MDO]_9$
M	AIBN-$[BCr]_5$-$[MDO]_{11}$-H
N	$[BCr]_8$-$[MDO]_8$
O	AIBN-$[BCr]_6$-$[MDO]_{10}$-H
P	$[BCr]_5$-$[MDO]_{12}$

Q	AIBN-[BCr]$_7$-[MDO]$_9$-H

3.5.4. BCr-MDO 75/25 by bulk copolymerization

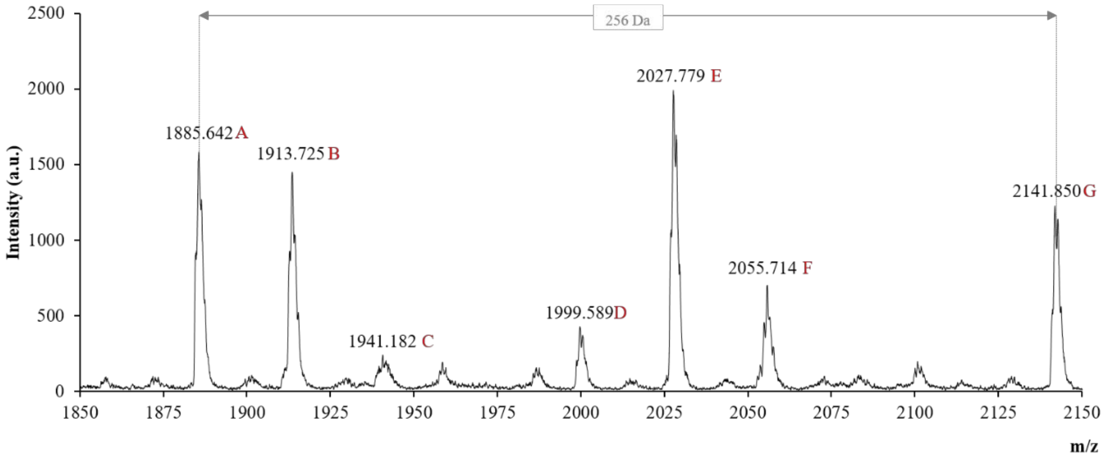

Figure S13. *Zoom of the MALDI top of the BCr-MDO 25/75 copolymer, presented in Figure 4b, for the identification of the peaks*

Table S5. *Identification of the peaks from Figure S13.*

Peak	Structure
A	AIBN-[BCr]$_7$-[MDO]$_7$-H
B	AIBN-[BCr]$_8$-[MDO]$_6$-H
C	AIBN-[BCr]$_9$-[MDO]$_5$-H
D	AIBN-[BCr]$_7$-[MDO]$_8$-H
E	AIBN-[BCr]$_8$-[MDO]$_7$-H
F	AIBN-[BCr]$_9$-[MDO]$_6$-H
G	AIBN-[BCr]$_8$-[MDO]$_8$-H

3.6. Fitting of the reactivity ratios and Mayo-Lewis equation

Figure S14 shows the evolution of the cumulative copolymer composition (with respect to BCr) over the total conversion during the experiments carried out *in situ* in the NMR device. The continuous lines present the predicted cumulative copolymer composition calculated for the estimated reactivity ratios. Figure S15 shows the Mayo-Lewis plot for the reactivity ratios that were estimated(r_{BCr} = 0.017 ± 0.007 and r_{MDO} = 0.105 ± 0.013).

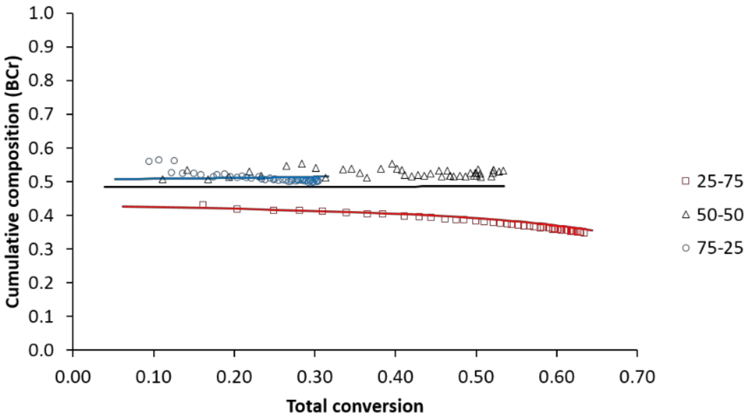

Figure S14. *Evolution of the cumulative copolymer composition over the total conversion. The open symbols are experimental point and the continuous line the fitting for the reactivity ratios.*

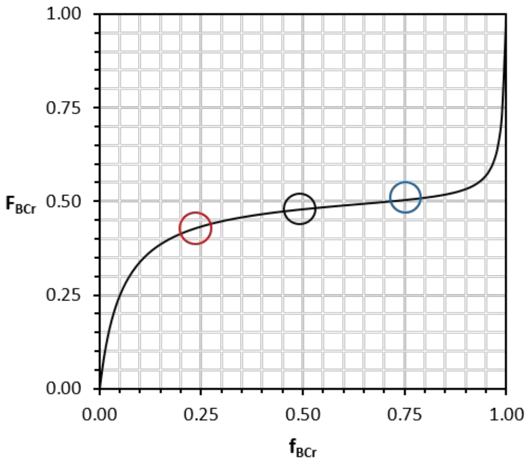

Figure S15. *Mayo-Lewis plot for the reactivity ratios estimated in this work for BCr and MDO (r_{BCr} = 0.017 ± 0.007 and r_{MDO} = 0.105 ± 0.013).*

3.7. Copolymerization of ECr and 2OCr with MDO

3.7.1. Copolymerization kinetics

Figure S16 shows the time evolution during *in situ* NMR copolymerization of ethyl crotonate and MDO (left) and 2-octyl crotonate and MDO (right). Figure S17 shows the time evolution of the open percentage of MDO during the copolymerization, with the copolymer of BCr-MDO (50/50) added as a reference. Last, Figure S18 and S19 show the ^{1}H-NMR spectra of the beginning and end of the copolymerization, with the assignation of each signal.

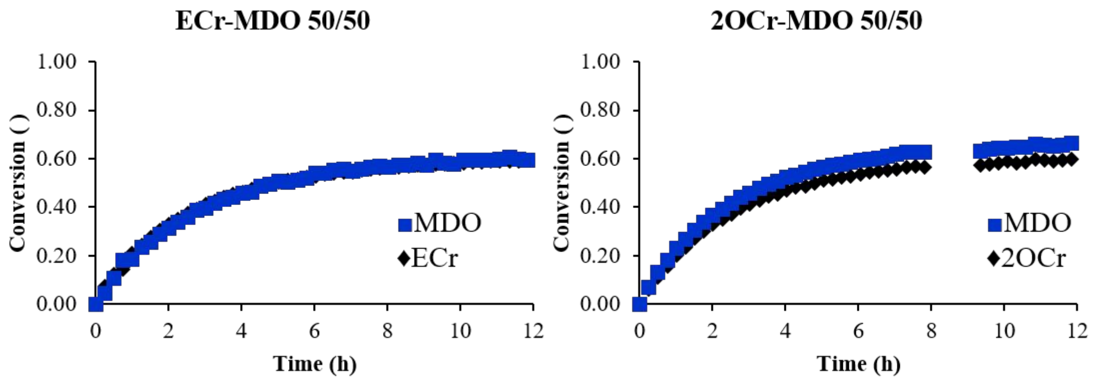

Figure S16. *Evolution of the copolymerization between ECr and MDO (left) and 2OCr and MDO (right).*

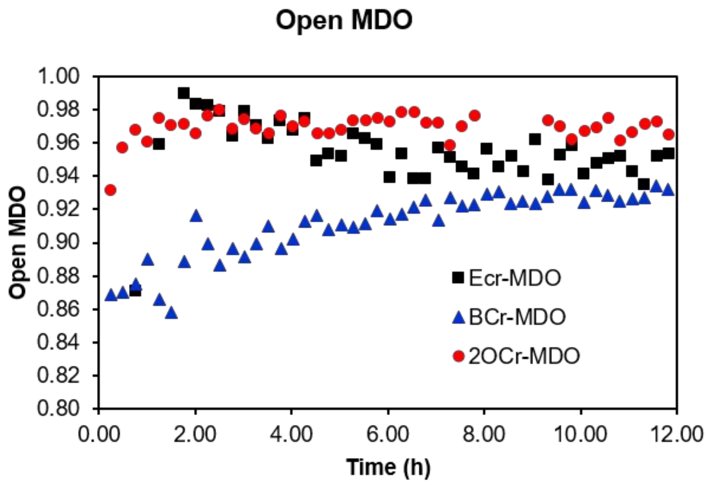

Figure S17. *Time evolution of the open MDO percentage during the in situ NMR kinetic experiments.*

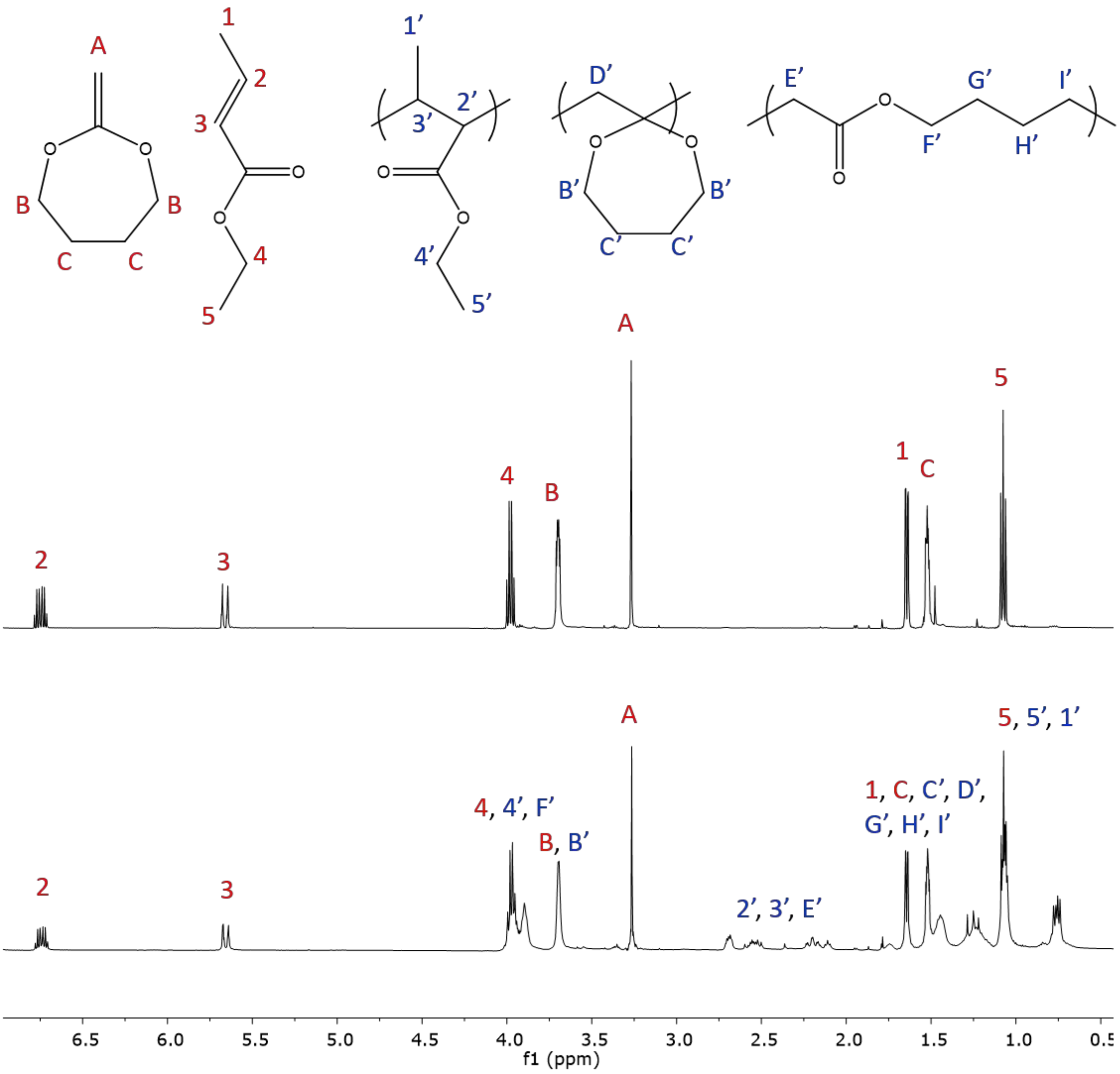

Figure S18. ^{1}*H-NMR of the copolymerization between ECr and MDO in a 50-50 mol ratio at the beginning of the reaction (top) and after 12 h (bottom).*

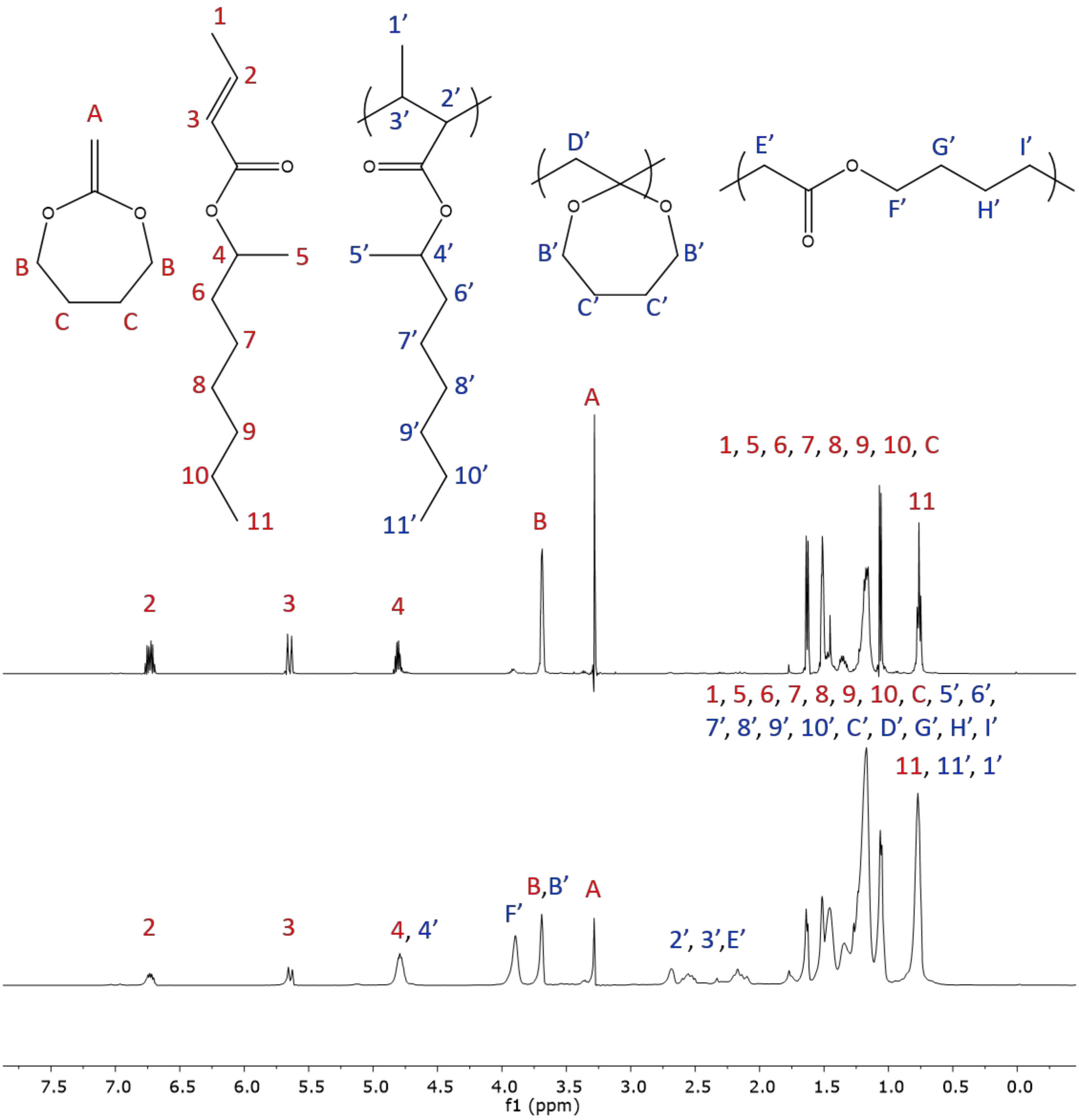

Figure S19. *^{1}H-NMR of the copolymerization between 2OCr and MDO in a 50-50 mol ratio at the beginning of the reaction (top) and after 12 h (bottom).*

3.7.2. Molar mass distribution of ECr and 2OCr copolymers

Table S6. *Weight average molar mass and dispersity of ECr-MDO and 2OCr-MDO copolymers.*

Copolymer	$\overline{M_w}$ (kg/mol)	Đ
ECr-MOD (50/50)	11.2	2.1
2OCr-MDO (50/50)	19.0	2.5

3.7.3. MALDI-TOF spectra of the ECr and 2OCr copolymers

Figure S20 and S21 show the MALDI-TOF spectra of the ECr-MDO and 2OCr-MDO copolymers, respectively.

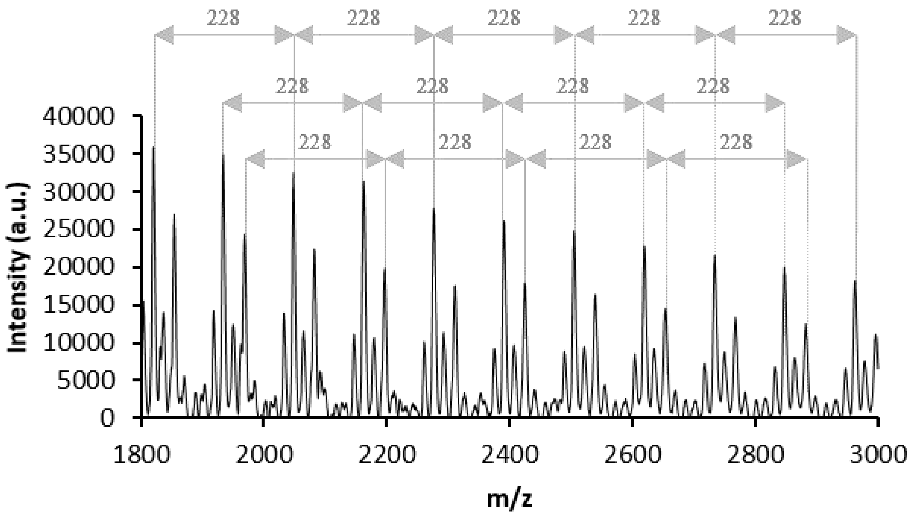

Figure S20. *MALDI-TOF spectrum of the ECr-MDO copolymer synthetized by solution polymerization in xylene, showing the alternating nature by the repetition of the 228 Da repeating unit.*

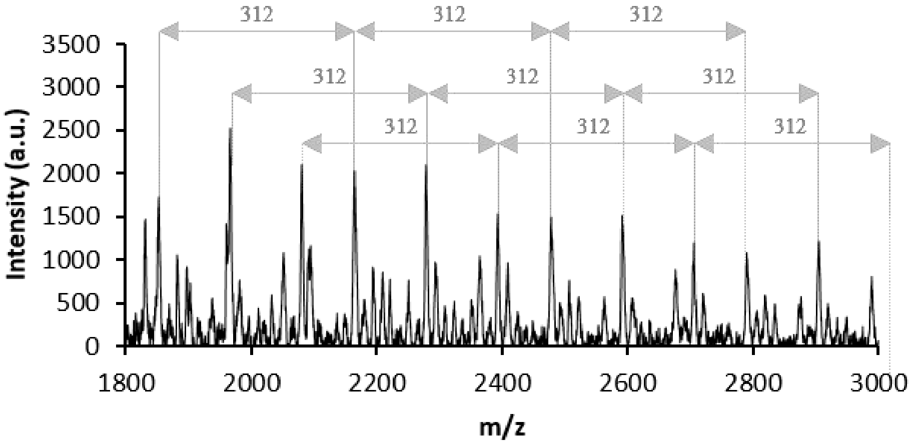

Figure S21. *MALDI-TOF spectrum of the 2OCr-MDO copolymer synthetized by solution polymerization in xylene, showing the alternating nature by the repetition of the 312 Da repeating unit.*

3.7.4. DSC traces of crotonate-MDO copolymers

Figure S22 presents the DSC traces of ECr-MDO (a), BCr-MDO (b) and 2OCr-MDO copolymers.

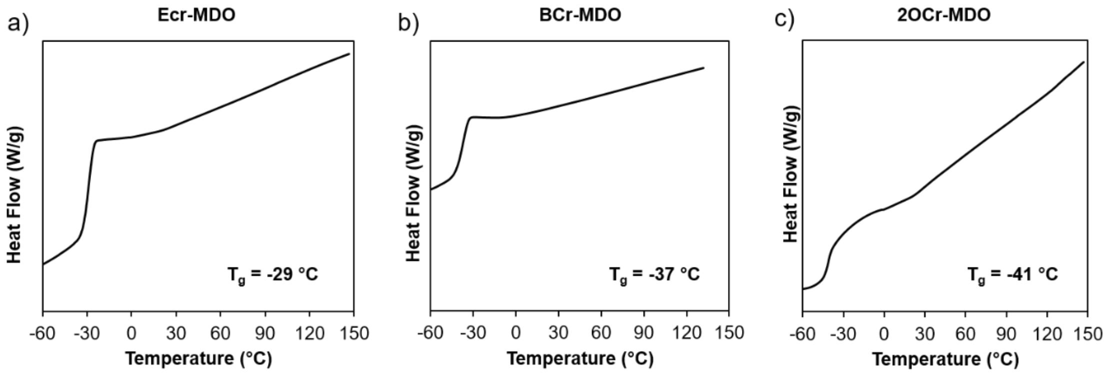

Figure S22. *DSC traces of ECr-MDO (a), BCr-MDO (b) and 2OCr-MDO copolymers*

3.7.5. Degradation experiments of the ECr and 2OCr copolymers

Figure S23 shows the molar mass distribution of the ECr and 2OCr right after the polymerization finished (in black) and after 2 h exposed to the KOH solution (red). As observed, most of the polymer distribution has molar masses below 2000 g/mol after degradation.

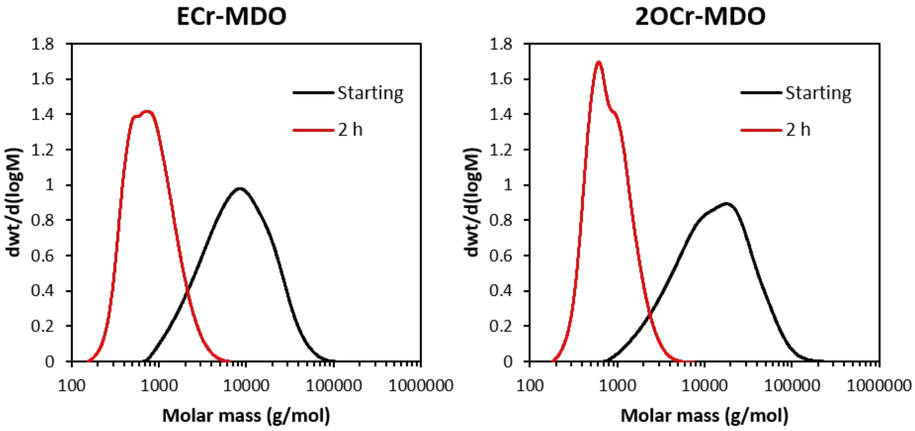

Figure S23. *Molar mass distribution of the ECr and 2OCr copolymers before and after degradation experiments.*

Additionally, the degradation products were analyzed by ^{1}H-NMR.

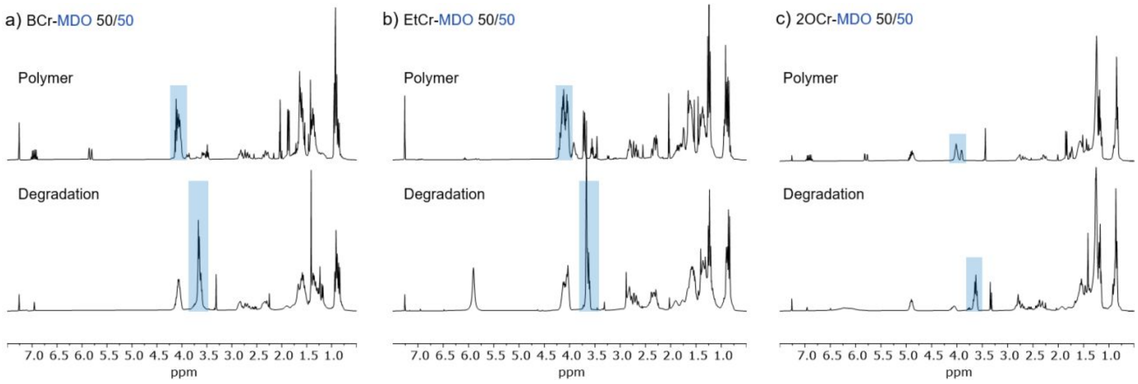

Figure S24. *^{1}H NMR of copolymers before and after degradation. Measured in $CDCl_3$ at 25 ^{o}C.*

Evidence of hydrolysis can be observed by ^{1}H-NMR spectroscopy. All copolymers show a characteristic signal at around 4.0-4.1 ppm corresponding to the methylene proton adjacent to the ester linkage. After subjecting the samples to hydrolysis, the corresponding degraded samples reveal an intensity decrease in the aforementioned methylene signal, and a new signal appears at around 3.6-3.7 ppm. Such a shift upfield would correspond to the hydrolyzed product, being the methylene adjacent to the alcohol group. This is supported also by ^{1}H-^{1}H COSY experiments (Figures S25-27 for BCr-MDO, ECr-MDO and 2OCr-MDO, respectively).

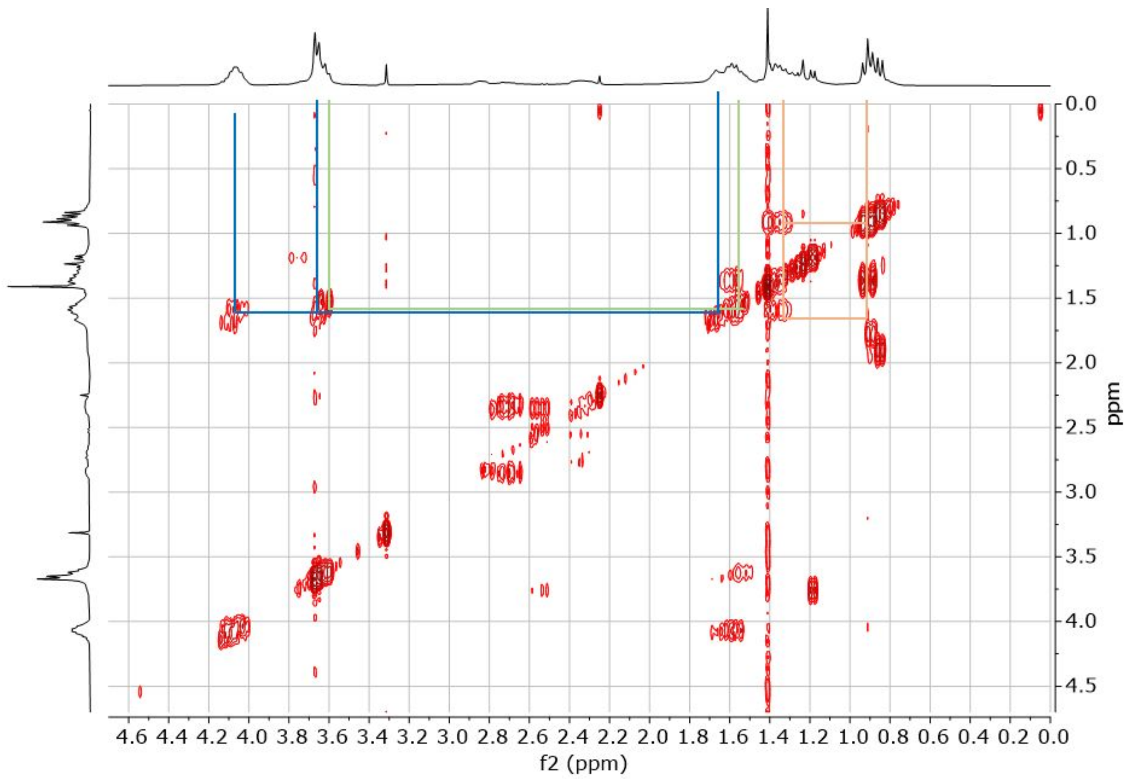

Figure S25. *^{1}H-^{1}H COSY of degraded BCr-MDO (50/50) copolymer. Measured in $CDCl_3$ at 25 ^{0}C.*

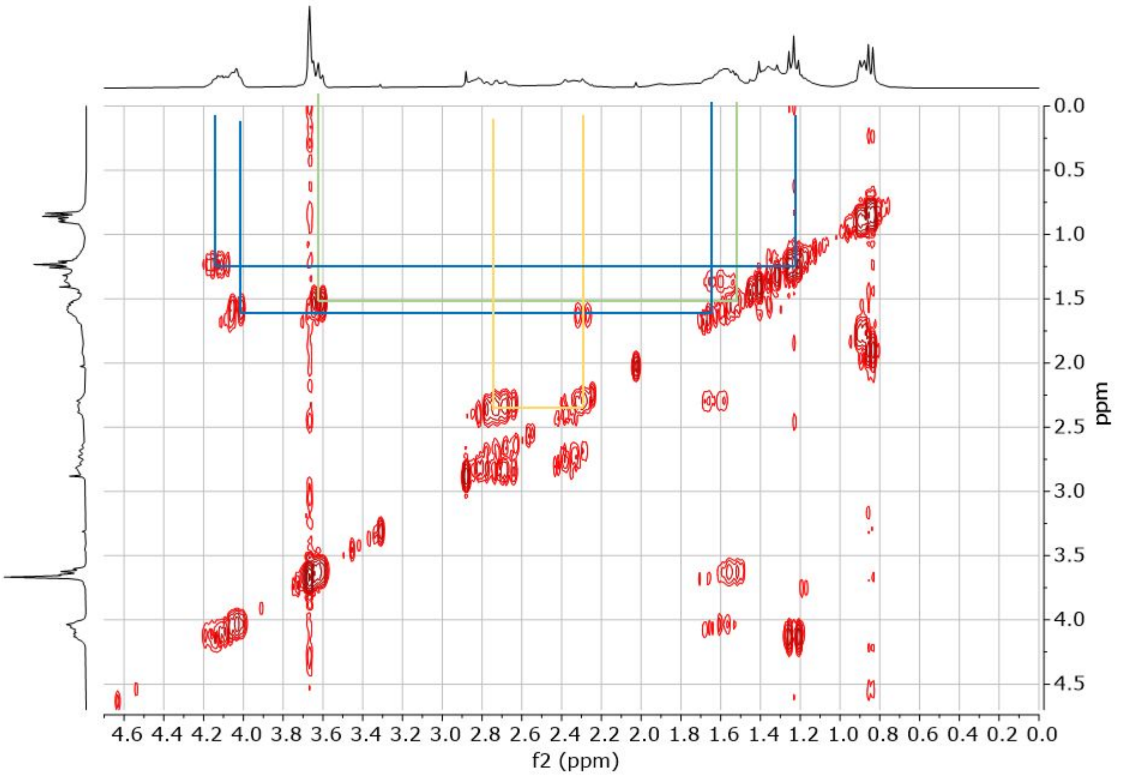

Figure S26. *^{1}H-^{1}H COSY of degraded ECr-MDO (50/50) copolymer. Measured in $CDCl_3$ at 25 ^{0}C.*

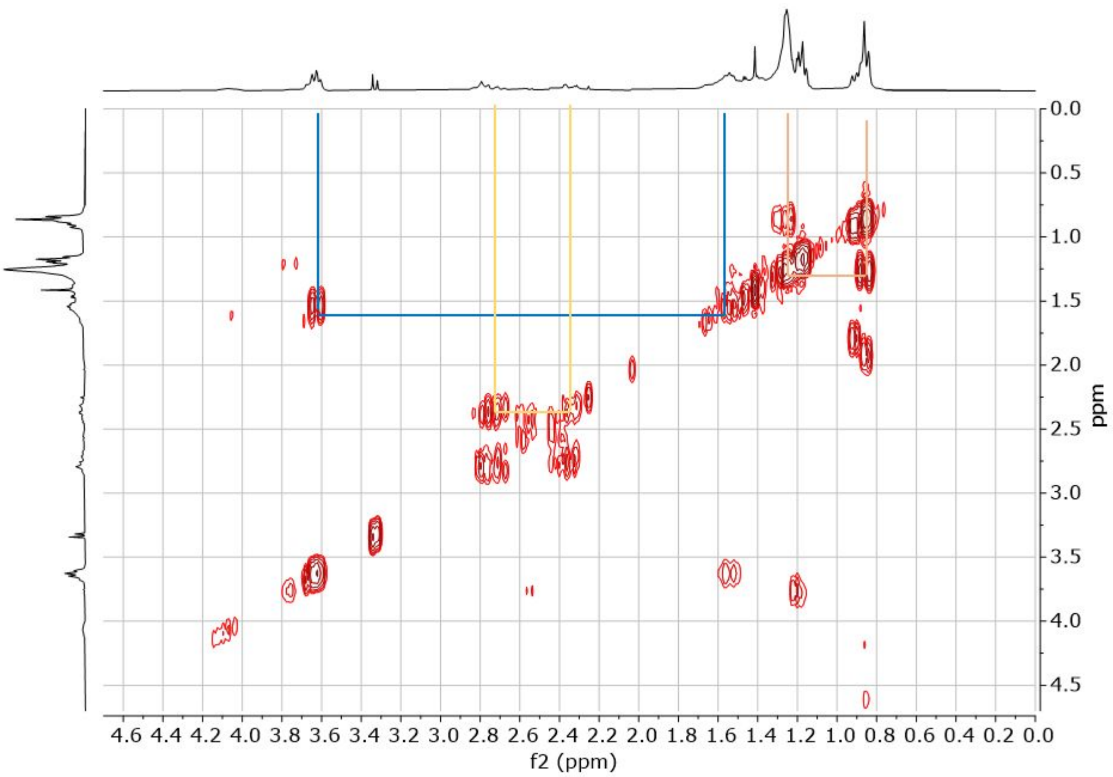

Figure S27. *^{1}H-^{1}H COSY of degraded 2OCr-MDO (50/50) copolymer. Measured in $CDCl_3$ at 25 ^{0}C.*
